# The ubiquitin ligase HERC1 regulates cell migration via RAF-dependent regulation of MKK3/p38 signaling

**DOI:** 10.1038/s41598-020-57756-7

**Published:** 2020-01-21

**Authors:** Leonardo Pedrazza, Taiane Schneider, Ramon Bartrons, Francesc Ventura, Jose Luis Rosa

**Affiliations:** Departament de Ciències Fisiològiques, Institut d’Investigació de Bellvitge (IDIBELL), Universitat de Barcelona, L’Hospitalet de Llobregat, Barcelona, Spain

**Keywords:** Cell signalling, Cell signalling, Stress signalling, Stress signalling

## Abstract

Protein modifications by phosphorylation or ubiquitylation have been selected throughout evolution as efficient regulatory mechanisms of cellular processes. Cell migration is a complex, highly coordinated process where these mechanisms must participate in an integrated manner to transmit signaling during migration. In this study, we show that the ubiquitin ligase HERC1 regulates the p38 signaling pathway, and that this regulation is mediated by the MAPK kinase MKK3. Moreover, we demonstrate a crosstalk between RAF and MKK3/p38 pathways where RAF acts upstream of MKK3. Mechanistically, HERC1 regulates the protein levels of C-RAF and MKK3. Thus, HERC1 ubiquitylates C-RAF, targeting it for proteasomal degradation, and RAF proteins regulate MKK3 mRNA levels. Accordingly, HERC1 knockdown induces C-RAF stabilization and activation of RAF proteins; in turn, this activation increases MKK3, which phosphorylates and activates p38. The importance of these observations is demonstrated by HERC1 regulation of cell migration through regulation of p38 signaling via a RAF-dependent mechanism. Thus, HERC1 plays an essential role as a regulator of crosstalk between RAF/MKK3/p38 signaling pathways during cell migration.

## Introduction

Post-translational modifications can modify proteins and regulate their functions. Protein ubiquitylation is a post-translational modification that covalently attaches ubiquitin to target proteins, altering protein function in an extraordinary variety of ways. This process occurs in three sequential steps: ubiquitin activation by a ubiquitin-activating enzyme (E1); transfer of activated ubiquitin from E1 to ubiquitin-conjugating enzyme (E2); and conjugation of ubiquitin to a lysine residue of a substrate protein by ubiquitin ligase enzyme (E3). This catalytic process can be repeated over several cycles, resulting in substrates modified on multiple lysine residues with different poly-ubiquitin chains. Since E3 ligases control substrate specificity and the ubiquitylation topology, they are emerging as key regulators of cellular processes^1–3^.

Cell migration plays a fundamental role in multiple physiological and pathological processes, including wound healing, embryogenesis, tissue morphogenesis, cancer metastasis, and inflammation. Numerous studies have demonstrated that mitogen-activated protein kinase (MAPK) pathways, including the Jun N-terminal kinase (JNK), p38, and extracellular signal-regulated protein kinase (ERK) signaling pathways, regulate cell migration via different mechanisms. Thus, the JNK signaling pathway modulates cell migration by phosphorylating proteins such as paxillin, DCX, Jun, and microtubule-associated proteins. Meanwhile, the p38 signaling pathway regulates migration by phosphorylating MAPK-activated protein kinase 2 (MAPKAP-K2 or MK2) and the ERK signaling pathway controls cell movement by phosphorylating myosin light chain kinase, calpain, or focal-adhesion kinase^4,5^.

Several ubiquitin E3 ligases also regulate cell migration by ubiquitylating the key proteins that control stress fiber formation, cell polarity, lamellipodial protrusions, and focal adhesion dynamics^6^. For example, E3 ligases Smurf1 and the Cul3/BACURD complex ubiquitylates RhoA to regulate stress fiber formation and cell polarity, while E3 ligase ASB2α ubiquitylates filamins to modulate the elasticity of the actin network, thus regulating cell spreading and cell migration. E3 ligases HACE1, XIAP, and SCF/FBX19 ubiquitylate Rac1, resulting in its degradation and suppressing lamellipodial protrusions. Conversely, sumoylation of Rac1 by the SUMO E3 ligase PIAS3 activates Rac1 to promote lamellipodial protrusions. Moreover, Smurf1-dependent ubiquitylation of TRAF4 also enhances Rac1 activation. Smurf1 and E3 ligase HECTD1 regulate focal adhesion assembly/disassembly through ubiquitylation of talin head and phosphatidylinositol 4-phosphate 5-kinase type I γ, respectively^6^.

Proteins containing HECT and RCC1-like domains form the HERC protein family, a subgroup of ubiquitin E3 ligases in the HECT family that regulate cellular processes such as cell cycle, cell signaling, DNA damage response, or antiviral response, and are implicated in cancer, neurological disorders, and other diseases^7^. Recently, it has been shown that the E3 ligase HERC1 regulates cell proliferation through control of the ERK signaling pathway targeting MAPK kinase kinase (MAPKKK) C-RAF for proteasomal degradation^8^. RAF (A-RAF, B-RAF, and C-RAF) serine/threonine kinases link RAS activation to activation of the MEK/ERK module. Through this mechanism, RAF kinases relay signals inducing cell proliferation, differentiation, and survival. C-RAF also plays an essential function in cell migration as a regulator of Rho downstream signaling^9^. Here, we show for the first time that the E3 ligase HERC1 regulates cell migration through activation of the MKK3/p38 module. More importantly, this activation is dependent on RAF proteins and involves an unexpected and unusual crosstalk between these MAPK pathways. Our study helps elucidate the complex regulation of cell migration, where ubiquitylation and phosphorylation mechanisms participate in an integrated manner to transmit signaling for cell mobilization.

## Results

### HERC1 regulates p38 signaling

**Figure 1:**
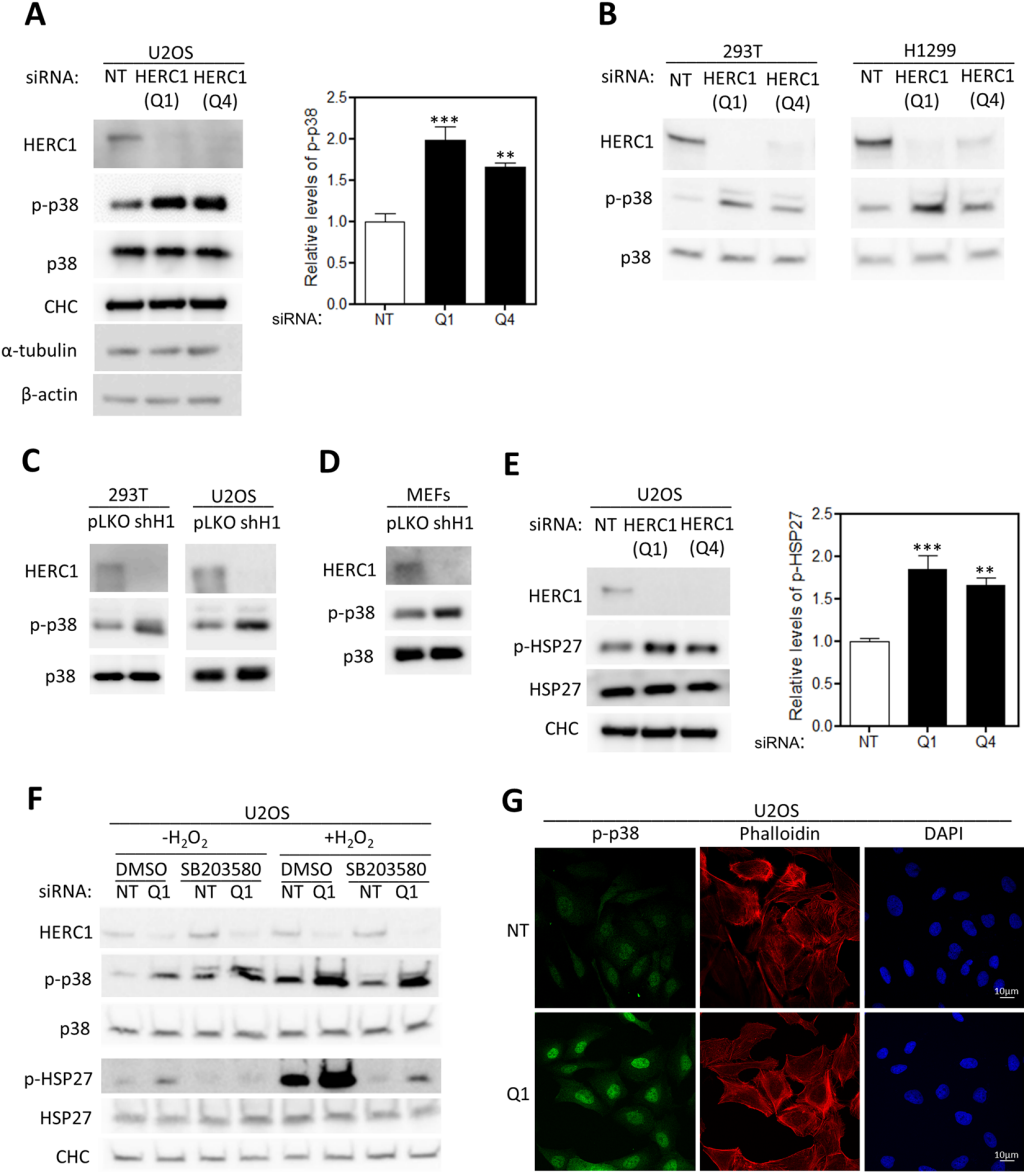
HERC1 regulates p38 signaling. (**A**) Lysates from U2OS cells transfected with NT or HERC1 (Q1 or Q4) siRNA were analyzed by immunoblotting with antibodies against the indicated proteins. Clathrin heavy chain (CHC) levels were used as protein load control. p-p38 levels were quantified and normalized by p38 levels (n = 4). **(B)** Lysates from HEK-293T or H1299 cells were analyzed as above. **(C)** HEK-293T or U2OS cells infected with lentivirus shH1 (HERC1 shRNA) or pLKO (the lentivirus plasmid vector as negative control) were analyzed by immunoblotting with antibodies against the indicated proteins. **(D)** MEF cells infected with lentivirus were analyzed as above. **(E)** Lysates from U2OS cells transfected with NT or HERC1 (Q1 or Q4) siRNA were analyzed as in (**A**). p-HSP27 levels were quantified and normalized by HSP27 levels (n = 4). **(F)** U2OS cells were transfected with NT or HERC1 siRNA. Seventy-two hours post-transfection, they were treated with the p38 kinase activity inhibitor, SB203580 (10 μM for 1 hour), and subsequently with hydrogen peroxide (H_2_O_2_) for 30 minutes. The cell lysates were analyzed as in (**A**). **(G)** U2OS cells transfected with NT or HERC1 (Q1) siRNA were stained for p-p38 (green), F-actin (red), and nuclei (blue) and analyzed by confocal microscopy. Data are expressed as mean ± S.E.M. Statistical analysis was carried out as described in “Materials and Methods”. Significant differences are relative to NT siRNA. **p < 0.01; ***p < 0.001.

We have previously shown that the ubiquitin E3 ligase HERC1 regulates the RAF/MEK/ERK signaling pathway^8^. We wondered whether HERC1 might also regulate other MAPK pathways. To answer this question, we decided to analyze the p38 MAPK pathway in HERC1-depleted human cells. Human osteosarcoma U2OS cells were transfected with small interfering RNA (siRNA), using two different HERC1 siRNAs (Q1 and Q4) and a non-targeting (NT) siRNA. With both HERC1 siRNAs, a significant increase in p38 phosphorylation (p-p38) was observed in HERC1-depleted cells (Fig. 1A). Under these conditions, p38 protein levels (p38) were not modified. Protein levels of Clathrin heavy chain (CHC), α-tubulin or β-actin were not modified either. Similar results were observed in other tumorigenic cells (H1299, a human non-small cell lung carcinoma cell line) and in non-tumorigenic cells (HEK-293T, a human embryonic kidney 293T cell line) (Fig. 1B). These results were also confirmed using short hairpin RNA (shRNA): HEK-293T and U2OS cells were infected with mock lentivirus or with lentivirus expressing shRNA against HERC1 (shH1) and an increase in p38 phosphorylation (p-p38) was observed in HERC1-depleted cells (Fig. 1C). Similar results were also obtained in mouse embryonic fibroblasts (MEFs) (Fig. 1D). To show that the increase in p38 phosphorylation correlates with an increase in its activation, we analyzed the phosphorylation of HSP27, a known downstream target of p38 activity^10^, and observed a marked increase in HSP27 phosphorylation in HERC1-depleted cells (Fig. 1E). Incubation with SB203580, an inhibitor of p38 activity, confirmed that HSP27 phosphorylation was dependent on p38 activity (Fig. 1F). These effects were intensified after stimulation of p38 activity with hydrogen peroxide (H_2_O_2_) and strongly inhibited in the presence of p38 inhibitor (Fig. 1F). p38 activation is also associated with its translocation to the nucleus. Immunofluorescence experiments showed an increase in p38 phosphorylation and its nuclear location in HERC1-depleted cells (Fig. 1G). In conjunction, these results demonstrate the involvement of the ubiquitin ligase HERC1 in the regulation of p38 signaling.

### HERC1 regulates p38 signaling through the MAPK kinase MKK3

**Figure 2:**
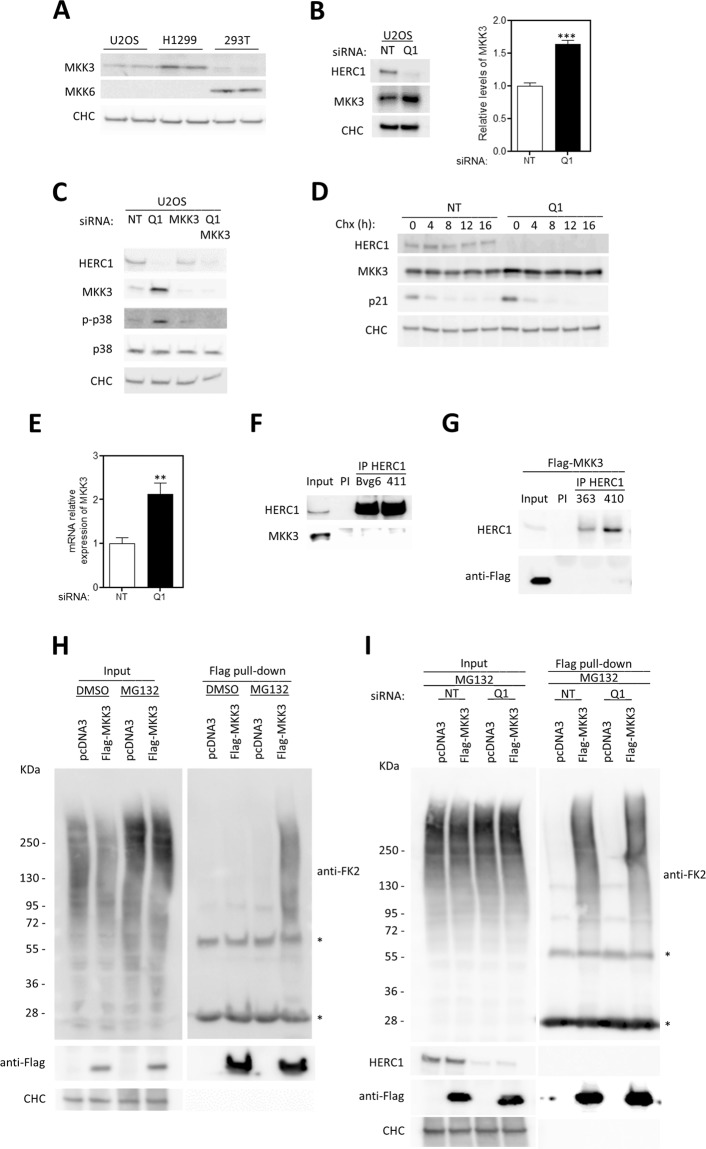
HERC1 regulates p38 signaling trough MKK3. (**A)** Two independent lysates for each human cell line (U2OS, H1299, and HEK-293T) were analyzed by immunoblotting to evaluate MKK3 and MKK6 protein expression. CHC levels were used as protein load control. **(B)** Lysates from U2OS cells transfected with NT or HERC1 (Q1) siRNA were analyzed by immunoblotting with antibodies against the indicated proteins. MKK3 levels were quantified and normalized by CHC levels (n = 4). **(C)** Lysates from U2OS cells transfected with NT, HERC1 (Q1), or MKK3 siRNA were analyzed by immunoblotting with antibodies against the indicated proteins. **(D)** U2OS cells were transfected with NT or HERC1 siRNA. Seventy-two hours post-transfection, cells were treated with cycloheximide for 0, 4, 8, 12, and 16 h. Time-course of MKK3 expression was analyzed by immunoblotting. CHC levels were used as protein load control and p21 levels as a validation control of the experiment. **(E)** U2OS cells transfected with NT or HERC1 (Q1) siRNA were analyzed by quantitative real-time PCR (RT-qPCR). MKK3 mRNA expression levels were quantified and the housekeeping gene TBP was used to normalize them (n = 8). **(F)** Supernatants (input) of lysates from HEK-293T cells were immunoprecipitated (IP) using anti-HERC1 antibodies (Bvg6 and 411) and analyzed by immunoblotting with antibodies against the indicated proteins. Pre-immune serum (PI) was used as a negative control. **(G)** Supernatants (input) of lysates from HEK-293T cells transfected with Flag-MKK3 were immunoprecipitated (IP) using anti-HERC1 antibodies (363 and 410) and analyzed as above. Pre-immune serum (PI) was used as a negative control. **(H)** U2OS cells transfected with pcDNA3 or Flag-MKK3 were incubated in the presence of the proteasome inhibitor MG132 (10 μM) for 6 hours. Control cells were incubated with dimethyl sulfoxide (DMSO). Flag pull-down was performed as indicated in “Materials and Methods”. Proteins retained in the resin were analyzed by immunoblotting with the indicated antibodies. **(I)** U2OS cells were transfected with NT or HERC1 siRNA. Twenty-four hours later, cells were transfected with pcDNA3 or Flag-MKK3 plasmids. Forty-eight hours later, Flag pull-down was performed as indicated in “Materials and Methods”. Inputs and pull-downs were analyzed as in (H). Statistical analysis was carried out as described in “Materials and Methods”. Significant differences are relative to NT siRNA. **p < 0.01; ***p < 0.001.

Activation of the p38 MAPK is mediated by its phosphorylation by a MAPK kinase (MAPKK or MKK). The MKKs mainly involved in p38 activation are MKK3 and MKK6^11^. Although, *in vitro*, MKK4 can also phosphorylate p38^12^, MKK4 is preferably an activator of JNK, and MKK3 and MKK6 are the predominant activators of p38^13^. MKK3 and MKK6 appear to have redundant functions during development, as MKK3 or MKK6 knockout mice are viable whereas double knockout mice died during gestation^13^. We analyzed expression of MKK3 and MKK6 isoforms in human cells. Immunoblot analysis using specific antibodies showed expression of MKK3 in U2OS and H1299 cell lines and of MKK6 in the HEK-293T cell line (Fig. 2A). Next, we focused on MKK3 expression in HERC1-depleted U2OS cells and observed a significant increase in MKK3 protein levels after HERC1 knockdown (Fig. 2B). Interestingly, the increase in p38 phosphorylation observed in HERC1-depleted cells was abolished by MKK3 depletion, showing that the increase in p-p38 was caused by MKK3 (Fig. 2C). Experiments in the presence of cycloheximide, a translation inhibitor, demonstrated that the increase in MKK3 was not due to an increase in the half-life of MKK3 (Fig. 2D). Analysis of mRNA levels showed an increase in MKK3 levels in HERC1-depleted cells (Fig. 2E). These data suggest that the increase in MKK3 protein levels by HERC1 depletion is caused by an increase in its mRNA levels.

The above results led us to analyze whether HERC1 and MKK3 could interact. Immunoprecipitation experiments using different anti-HERC1 antibodies failed to detect interaction between HERC1 and MKK3 (both endogenous MKK3 and transfected Flag-MKK3) proteins (Fig. 2F,G).

Because HERC1 is a ubiquitin ligase, we wondered whether HERC1 might be regulating MKK3 ubiquitylation. To determine this, we first analyzed MKK3 ubiquitylation in the absence and presence of the proteasome inhibitor MG132. U2OS cells were transfected with constructs expressing Flag-MKK3 or the negative control pcDNA3. Forty-eight hours later, lysates from these cells were pulled down using Flag resin. Inputs and pull-down proteins were analyzed by PAGE/SDS and immunoblotted with the anti-ubiquitylated proteins antibody (FK2) or with specific antibodies against the proteins indicated (Fig. 2H). An increase in polyubiquitylated Flag-MKK3 was detected with the FK2 antibody in the presence of MG132 (Fig. 2H, lane 8 compared with lane 6), indicating MKK3 regulation by the proteasome. Next, we analyzed the role of HERC1 in MKK3 ubiquitylation. U2OS cells were transfected with HERC1 or non-targeting (NT) siRNAs. Twenty-four hours later, cells were also transfected with plasmids expressing Flag-MKK3 or pcDNA3 as negative control. Forty-eight hours later, lysates were prepared for pull-down experiments. Inputs and pull-downs were analyzed by PAGE/SDS and immunoblotting (Fig. 2I). We observed that the ubiquitylation detected with the FK2 antibody remained unchanged after HERC1 knockdown (Fig. 2I, lane 8 compared with lane 6). These data suggest that MKK3 ubiquitylation is not regulated by HERC1.

### RAF-dependent regulation of MKK3/p38 signaling by HERC1

**Figure 3:**
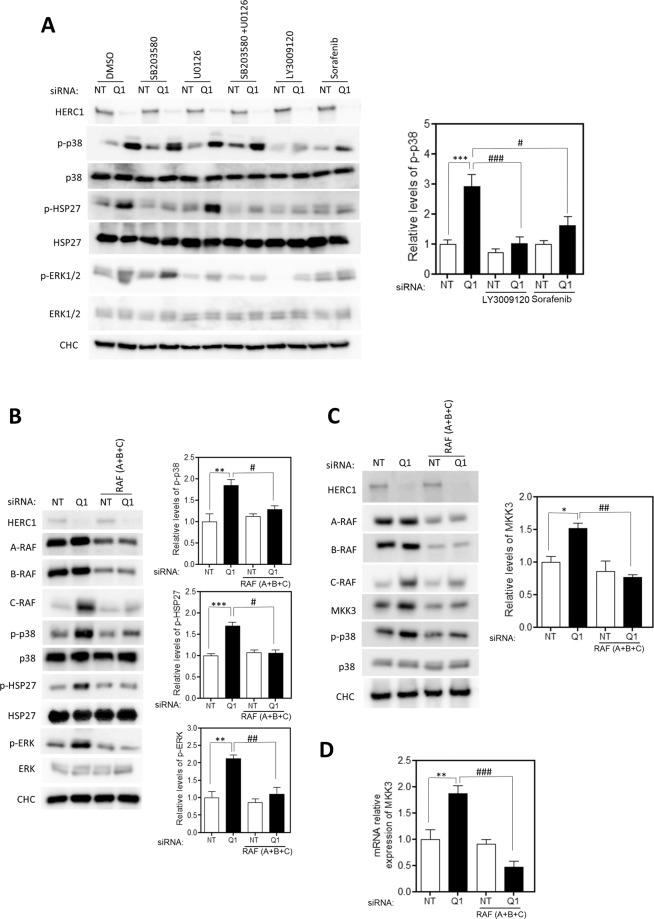
RAF-dependent regulation of MKK3/p38 signaling by HERC1. (**A)** U2OS cells were transfected with NT or HERC1 (Q1) siRNA. Seventy-two hours post-transfection, cells were treated with 10 μM of SB203580, U0126, LY3009120, or Sorafenib for 1 hour. Control cells were incubated with dimethyl sulfoxide (DMSO). Lysates from U2OS cells were analyzed by immunoblotting. p-p38 levels were quantified and normalized by p38 levels (n = 4). **(B)** Lysates from U2OS cells transfected with the indicated siRNA (NT: non-targeting; Q1: HERC1; A: A-RAF; B: B-RAF; and C: C-RAF) were analyzed by immunoblotting with antibodies against the indicated proteins. **(C)** Lysates from U2OS cells transfected with the indicated siRNA (NT: non-targeting; Q1: HERC1; A: A-RAF; B: B-RAF; and C: C-RAF) were analyzed by immunoblotting with antibodies against the indicated proteins. MKK3 levels were quantified and normalized by CHC levels (n = 4). **(D)** U2OS cells transfected with the indicated siRNA (NT: non-targeting; Q1: HERC1; A: A-RAF; B: B-RAF; and C: C-RAF) were analyzed by RT-qPCR. MKK3 mRNA expression levels were quantified and levels of the housekeeping gene TBP were used to normalize them (n = 8). Data are expressed as mean ± S.E.M. Statistical analysis was carried out as described in “Materials and Methods”. * or ^#^p < 0.05; ** or ^##^p < 0.01; *** or ^###^p < 0.001.

MAPK signaling pathways are organized in modular cascades in which activation of upstream kinases leads to sequential activation of a MAPK module. Although MAPK pathways are depicted as linear signaling pathways, in some situations crosstalk between MAPK signaling pathways has been reported^14^. The observation that HERC1 regulates the RAF/MEK/ERK module^8^ led us to ask whether this module could be involved in p38 activation. To test this, we decided to use specific inhibitors for some of these kinases. We observed that in the presence of U0126, a highly selective inhibitor of MEK activity, MKK3 (p-p38) and p38 (p-HSP27) activation by HERC1 knockdown was not inhibited, whereas ERK phosphorylation (p-ERK) was impaired (Fig. 3A, lanes 5–6 compared with 1–2). In the presence of the inhibitor of p38 activity, ERK activation (p-ERK) by HERC1 knockdown was not inhibited, whereas p38 activity (p-HSP27) was impaired (Fig. 3A, lanes 3–4 compared with 1–2). Notably, in the presence of the inhibitor of RAF activity (LY3091020), MKK3 and p38 activation was strongly inhibited (Fig. 3A, lanes 9–10 compared with 1–2). As expected, under these conditions ERK activation was inhibited (Fig. 3A, lanes 9–10 compared with 1–2). We confirmed these results using another inhibitor of RAF activity (Sorafenib), observing decreased p38 activation in the presence of Sorafenib (Fig. 3A, lanes 11–12 compared with 1–2). Interestingly, this inhibition of p38 activation was higher with LY3091020 than with Sorafenib (Fig. 3A), probably due to the different specificities of these inhibitors: Sorafenib is an inhibitor of B-RAF and C-RAF isoforms, while LY3091020 inhibits all RAF isoforms^15,16^.

The above experiments show that activation of MKK3/p38 signaling by HERC1 knockdown was dependent on RAF activity. Notably, these data indicate a crosstalk between RAF and p38 signaling pathways. To confirm these results using another experimental approach, we decided to perform knockdown experiments of the three isoforms of RAF (A-RAF, B-RAF, and C-RAF). The MKK3 and p38 activation observed in HERC1-depleted cells was inhibited by knockdown of RAF isoforms (Fig. 3B).

### HERC1 regulates RAF-dependent MKK3 expression

The increase in MKK3 protein levels in HERC1-depleted cells led us to analyze whether this increase was mediated by RAF proteins. We observed that knockdown of RAF isoforms abolished the increase in MKK3 in HERC1-depleted cells (Fig. 3C). In agreement with this, p38 phosphorylation was also suppressed (Fig. 3C). An analysis of mRNA levels showed that the increase in MKK3 expression in HERC1-depleted cells was abolished by RAF knockdown (Fig. 3D). In conjunction, these results suggest that MKK3 expression is regulated by HERC1 via a RAF protein-dependent mechanism.

### HERC1 regulates cell migration

**Figure 4:**
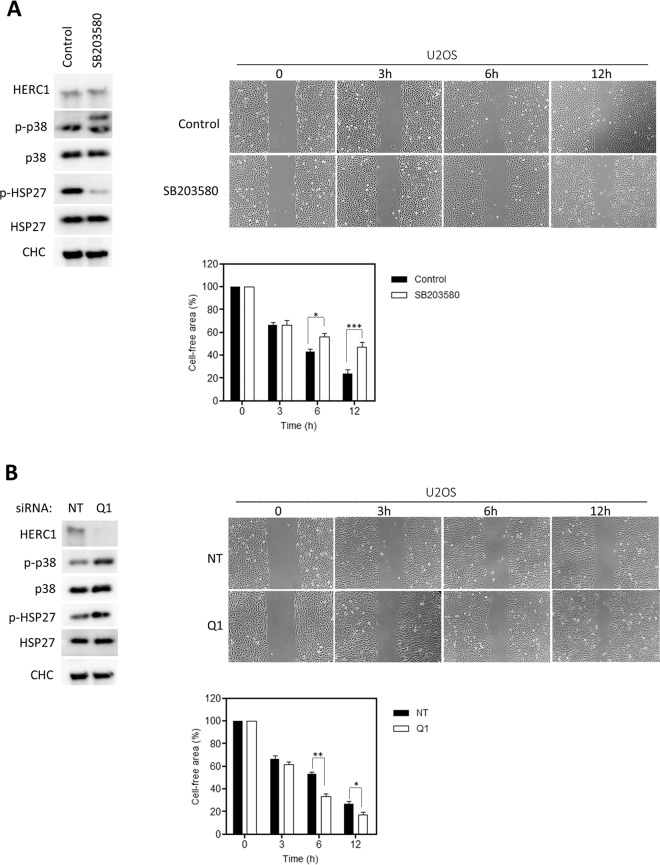
HERC1 regulates cell migration. (**A**) *In vitro* wound healing assay of U2OS cells (magnification x100). Cells were seeded onto 6-well cell culture plates and cultured to confluency. Cells were non-treated (control) or treated with 10 μM of SB203580 for 1 hour. Subsequently, a cell-free area was created (linear wound) using a sterilized 10 μL tip. Cell migration into the wound area was monitored. Representative time-lapse microscopy snapshots at specific time points (0, 3, 6, 12 h) were used to compare cell migration between groups (n = 4). **(B)** U2OS cells were transfected with NT or HERC1 (Q1) siRNA. Seventy-two hours post-transfection, an *in vitro* wound healing assay was performed as indicated above. Data are expressed as mean ± S.E.M. Statistical analysis was carried out as described in “Materials and Methods”. *p < 0.05; **p < 0.01; ***p < 0.001.

MAPKs play crucial roles in cell migration, a process that is central to embryonic development, plays a major role in cancer invasion and metastasis, and is known to involve ubiquitylation^4,6,17^. Cell migration can be analyzed through a wound healing assay in which a cell-free area is created in a confluent monolayer by physical exclusion or by removing the cells from the area. Exposure to the cell-free area induces cells to migrate into the gap. In our experiments, the monolayer was scratched with a pipette tip and migration into the gap was imaged over 12 hours using a transmitted light microscope. Images were analyzed and the cell-free area represented (Fig. 4A, Control). In this model, we studied whether cell migration was dependent on p38 activity. In the presence of an inhibitor of p38 activity, a significant delay in wound healing was observed at 6 and 12 hours (Fig. 4A, SB203580).

**Figure 5:**
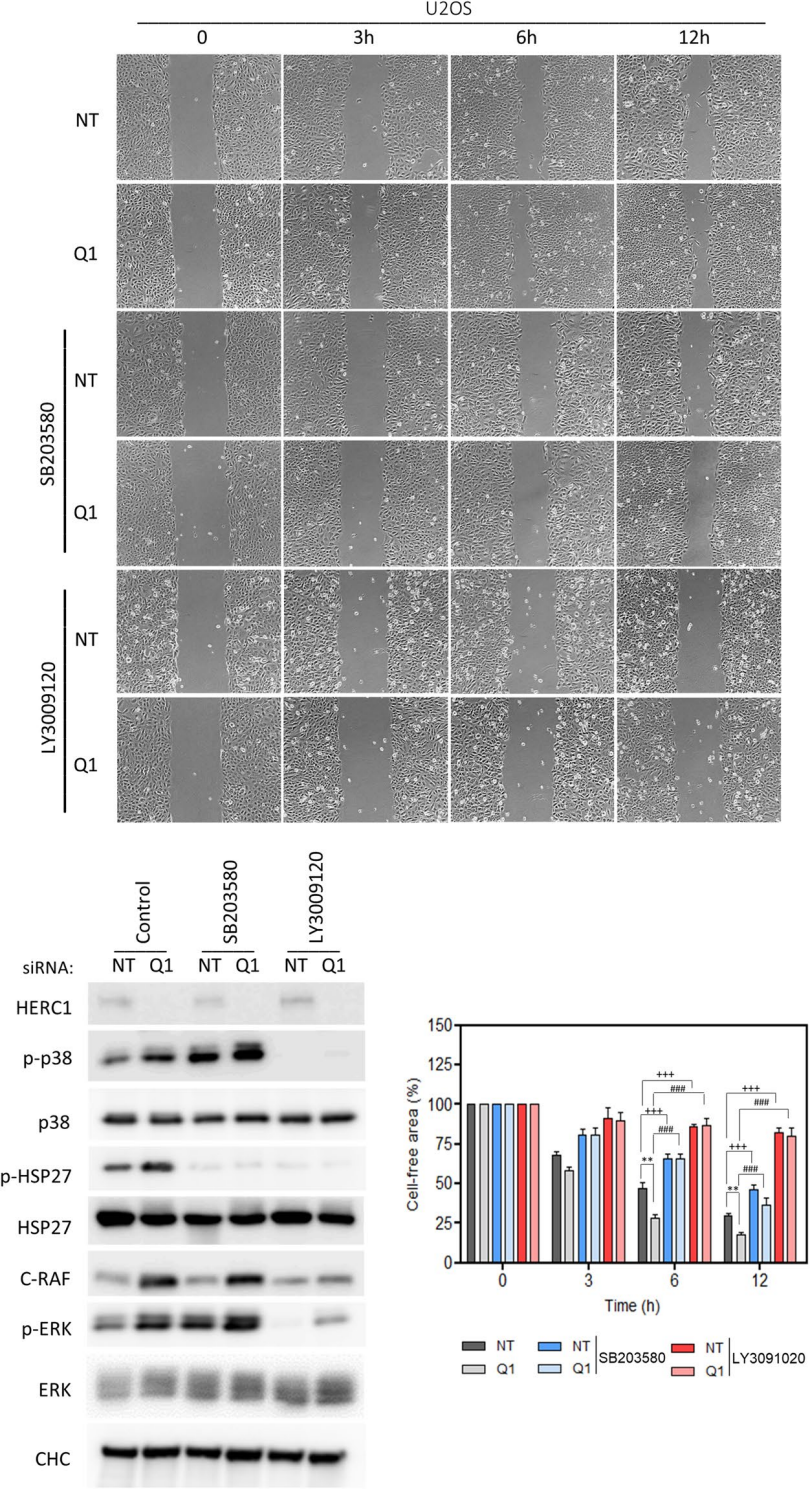
RAF activity-dependent regulation of cell migration by HERC1. U2OS cells were transfected with NT or HERC1 (Q1) siRNA for seventy-two hours. Cells cultured to confluency were non-treated or treated with 10 μM of SB203580 or LY3009120 for 1 hour. Next, an *in vitro* wound healing assay was performed as indicated in Fig. 4. Representative time-lapse microscopy snapshots at specific time points (0, 3, 6, 12 h) were used to compare cell migration between groups (n = 4). Percentages of cell-free area are expressed as mean ± S.E.M. Statistical analysis was carried out as described in “Materials and Methods”. **p < 0.01 represents differences relative to NT siRNA. ^+++^p < 0.001 represents differences between NT siRNA non-treated and treated with SB203580 or LY3009120 at the same time point. ^###^p < 0.001 represents differences between Q1 siRNA non-treated or treated with SB203580 or LY3009120, at the same time point.

Because cell migration is regulated by p38 activity and HERC1 regulates p38 activity (Fig. 1), we wondered whether HERC1 might be regulating cell migration. To test this, we performed wound healing assays in HERC1-depleted U2OS cells. We observed a significant increase in wound healing at 6 and 12 hours in HERC1-depleted cells (Fig. 4B). To determine whether this HERC1 regulation of cell migration was mediated by p38 activity, we performed wound healing assays in the presence of an inhibitor of p38 activity. We found that the increase in wound healing observed at 6 and 12 hours in HERC1-depleted cells was inhibited in the presence of the p38 inhibitor (Fig. 5, compare Q1 with Q1 + SB conditions).

### RAF-dependent regulation of cell migration by HERC1

Since HERC1 regulation of p38 activity was dependent on RAF activity (Fig. 3), we decided to study whether HERC1 regulation of cell migration was also dependent on RAF activity. Thus, we performed wound healing assays in the presence of an inhibitor of pan-RAF activity. We observed that cell migration was dependent on RAF activity (Fig. 5, compare NT with NT + LY conditions) and that the increase in wound healing at 6 and 12 hours in HERC1-depleted cells was strongly inhibited in the presence of the RAF inhibitor (Fig. 5, compare Q1 with Q1 + LY conditions).

**Figure 6:**
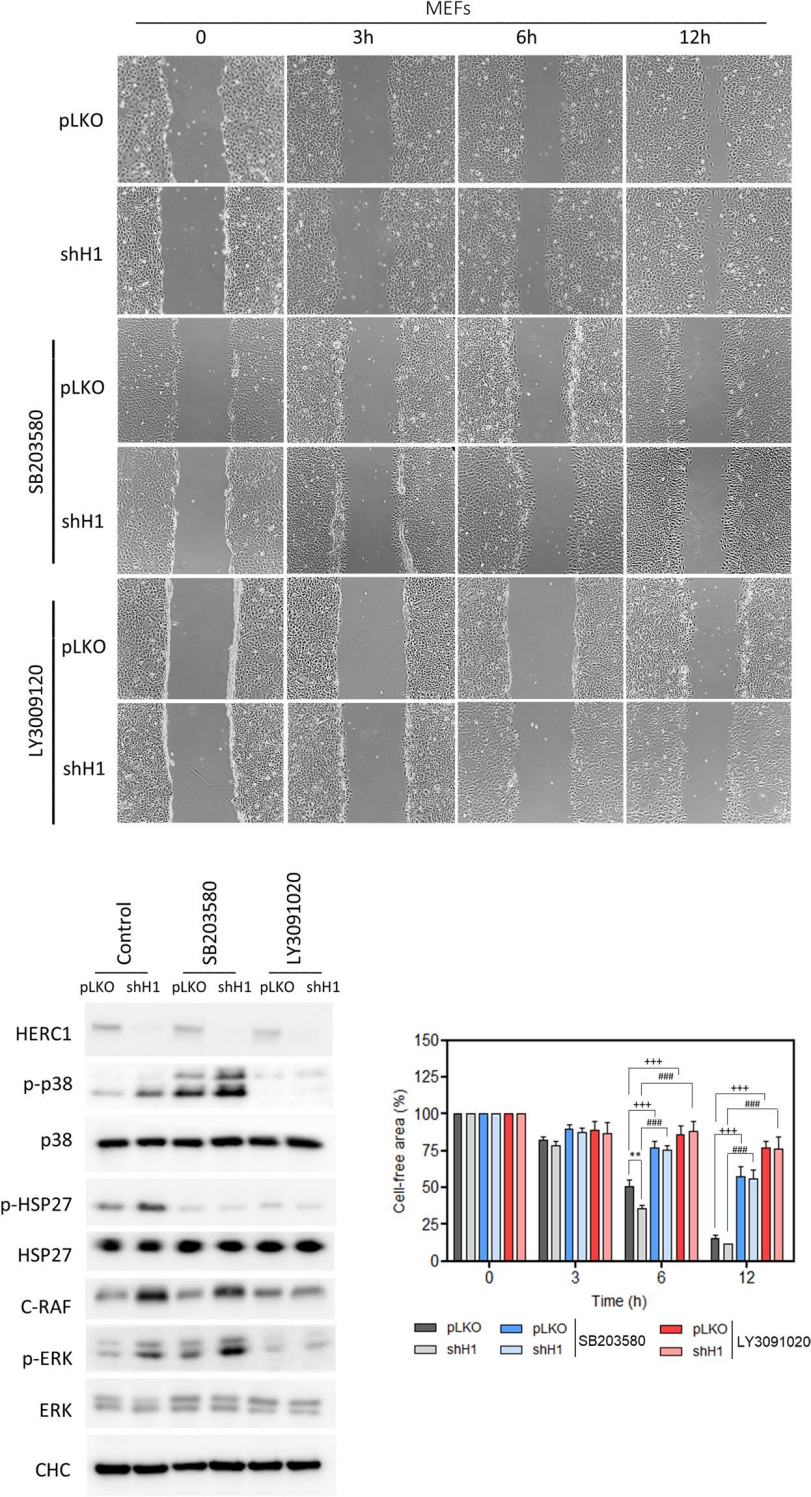
Regulation of cell migration by HERC1 in mouse embryonic fibroblasts (MEFs). MEFs were infected with lentivirus shH1 (HERC1 shRNA) or pLKO (the lentivirus plasmid vector as negative control). After selection of infected cells, an *in vitro* wound healing assay was performed as described in Fig. 4 (n = 4). Percentages of cell-free area are expressed as mean ± S.E.M. Statistical analysis was carried out as described in “Materials and Methods”. **p < 0.01 represents differences relative to pLKO. ^+++^p < 0.001 represents differences between pLKO non-treated and treated with SB203580 or LY3009120, at the same time point. ^###^p < 0.001 represents differences between shH1, non-treated and treated with SB203580 or LY3009120, at the same time point.

We analyzed whether the above results obtained in human osteosarcoma cells were maintained in other species. To this end, we performed wound healing assays in mouse embryonic fibroblasts (MEFs). First, we found that cell migration in these mouse cells was regulated by p38 and RAF proteins (Fig. 6, compare pLKO control with pLKO + SB conditions, and pLKO control with pLKO + LY conditions, respectively). Next, MEFs were infected with lentivirus expressing shRNA against HERC1 (shH1) and we found an increase in wound healing in HERC1-depleted cells (Fig. 6, compare pLKO with shH1 conditions). Under these conditions, regulation of cell migration by HERC1 knockdown was inhibited in the presence of the p38 inhibitor (Fig. 6, compare shH1 with shH1 + SB conditions) and the RAF inhibitor (Fig. 6, compare shH1 with shH1 + LY conditions). An immunoblot analysis demonstrated that p38 was activated in HERC1-depleted MEFs and that the presence of the RAF inhibitor was sufficient to abrogate this activation (Fig. 6).

**Figure 7:**
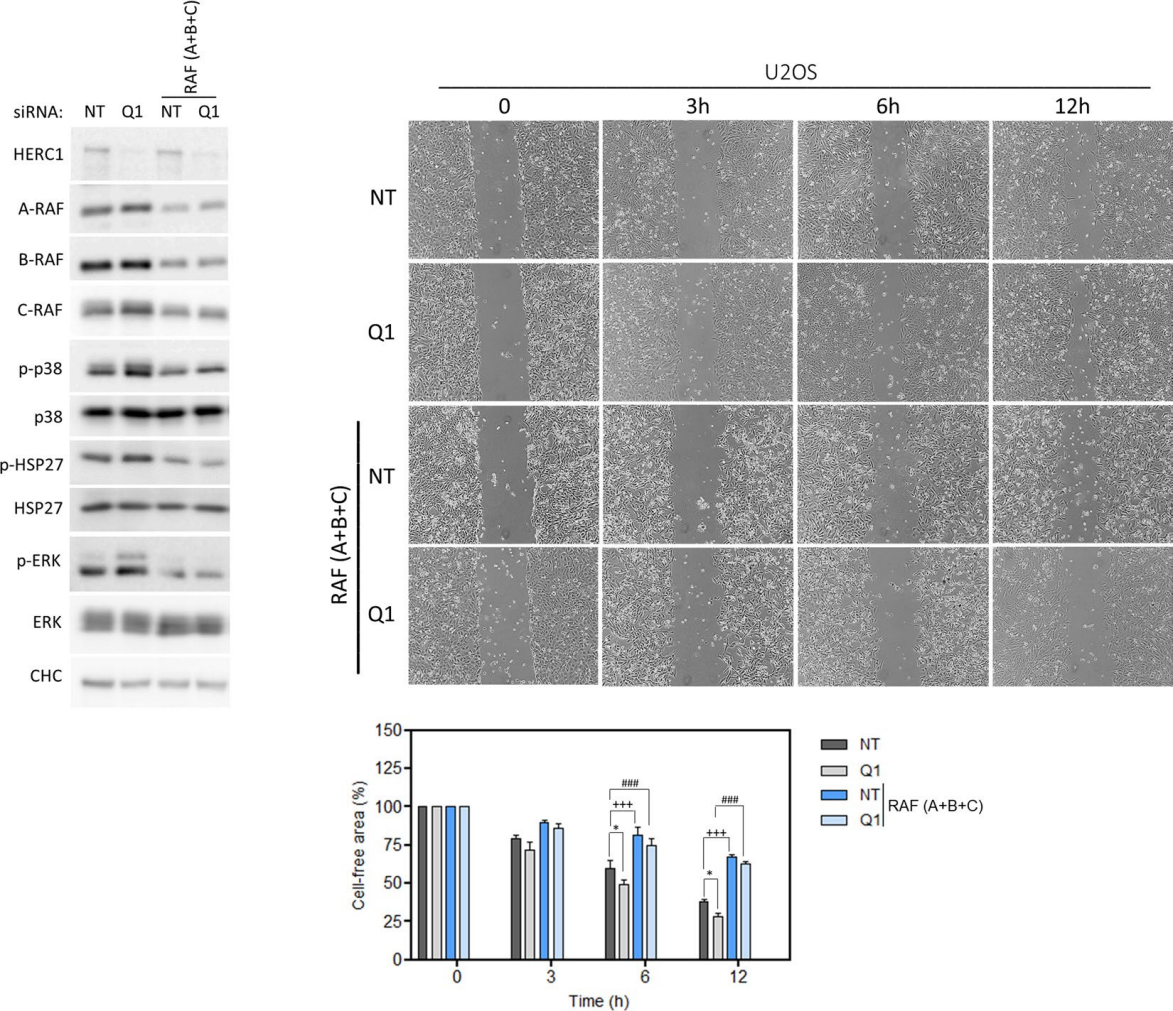
RAF-dependent regulation of cell migration by HERC1. U2OS cells were transfected with the indicated siRNA (NT: non-targeting; Q1: HERC1; A: A-RAF; B: B-RAF; and C: C-RAF). Seventy-two hours post-transfection, an *in vitro* wound healing assay was performed as indicated above. Percentages of cell-free area are expressed as mean ± S.E.M. Statistical analysis was carried out as described in “Materials and Methods”. *p < 0.05 represents differences relative to NT siRNA. ^+++^p < 0.001 represents differences between NT siRNA and NT + RAF (A + B + C) siRNAs at the same time point. ^###^p < 0.001 represents differences between Q1 siRNA and Q1 + RAF (A + B + C) siRNAs, at the same time point.

Together, the above results demonstrate the regulation by HERC1 of cell migration in a RAF-dependent manner. To confirm these data, we tested a rescue experiment using the HERC1 cDNA but we could not observe a significant expression of this cDNA probably due to its large size (15 kbp for coding a protein of 532 kDa). The use of plasmids expressing different regions (1–1334 and 1–2958 amino acid residues) of HERC1, including C-RAF binding site, was not enough to modify cell migration regulated by HERC1 knockdown. As an alternative, we performed knockdown experiments of the three isoforms of RAF. The increase in wound healing observed at 6 and 12 hours in HERC1-depleted U2OS cells was rescued by knockdown of RAF isoforms (Fig. 7).

## Discussion

HERC1 has previously been characterized as a ubiquitin E3 ligase regulating the ERK signaling pathway in cell proliferation^8^. Here, we provide the first evidence that HERC1 also acts as a regulator of the p38 signaling pathway, and that this regulation is mediated by the MAPK kinase MKK3. Notably, we demonstrate an unexpected crosstalk between RAF and MKK3/p38 pathways, in which RAF proteins act upstream of MKK3 protein and HERC1 regulates RAF activity. The importance of this crosstalk and its regulation by HERC1 is highlighted during cell migration. These functions are evolutionarily conserved as HERC1 and RAF work similarly in mouse cells. In summary, our results reveal an unknown role of HERC1 in the regulation of cell migration, acting as a regulator of the crosstalk between RAF/MKK3/p38 signaling pathways.

**Figure 8:**
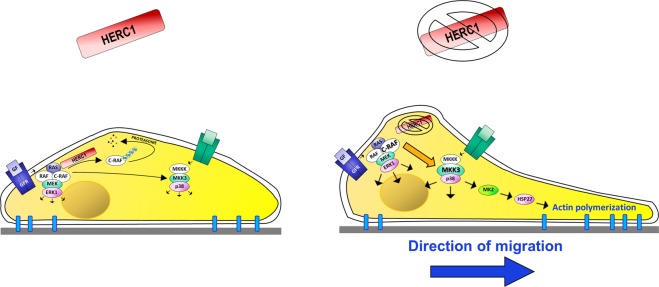
Working model for the regulation of cell migration by the ubiquitin ligase HERC1. RAS activation by external stimuli provides the anchor for homo- or heterodimerization of RAF proteins (A-RAF, B-RAF, and C-RAF). The activated dimer then phosphorylates and activates the downstream kinase MEK (MEK1/2), which in turn phosphorylates and activates ERK (ERK1/2). HERC1 plays a role in this signaling pathway by regulating C-RAF protein levels. Stress and other stimuli activate the MKKK/MKK3/p38 cascade. HERC1 knockdown increases C-RAF and MKK3 levels, resulting in ERK and p38 activation, which increases cell migration.

The E3 ligase HERC1 polyubiquitylates C-RAF, labeling it for proteasomal degradation^8^ and thus regulating C-RAF proteostasis. C-RAF is one of the three RAF family isoforms (A, B, and C) in humans. This family acts on the ERK signaling pathway as the first kinase (MAPKKK) of the cascade of this module (RAF/MEK/ERK). Stimuli that activate the RAS GTPase activate homodimers and/or heterodimers of the RAF proteins that phosphorylate and activate MEK (MEK1/2), which in turn phosphorylates and activates ERK (ERK1/2). Thus, ERK can transmit the stimulus signal phosphorylating multiple substrates that act on numerous cellular processes. HERC1 plays a role in this signaling pathway by regulating C-RAF protein levels (Fig. 8). Another MAPK signaling pathway is the p38 signaling pathway. Thus, a MAPKKK activated by stress stimuli phosphorylates and activates MKK3/MKK6, and these phosphorylate and activate p38. Like ERK, p38 phosphorylates multiple substrates that act on different cellular processes. Although MAPK pathways are depicted as linear signaling pathways, crosstalk between MAPK signaling pathways has been observed in some situations^18^. Here, we demonstrate that HERC1 regulates the p38 signaling pathway. Thus, HERC1 depletion rendered it possible to observe p38 activation in an MKK3-dependent manner (MKK6 was not detected in U2OS cells), and we found that under these conditions, MKK3 levels increased, which might suggest that MKK3 was the substrate for HERC1 ubiquitylation; however, association and ubiquitylation experiments showed that this was not the case. An analysis of MKK3 mRNA levels demonstrated a correlation with its protein levels, suggesting the involvement of a pre-translational regulation mechanism. The use of specific inhibitors or of siRNA against RAF isoforms showed that this activation of MKK3 and p38 was dependent on RAF proteins. In conjunction, this study demonstrates a crosstalk between RAF and MKK3 regulated by HERC1 (Fig. 8). Although we have not studied, we cannot discard a possible regulation of p38 signaling by other E3 ligases regulating stability and activity of C-RAF like X-linked and cellular IAPs^19^ or HUWE1^20^.

It has previously been shown that the specific antibody for the phosphorylated form of p38 used here recognizes two bands in U2OS cells, where the lower band corresponds to the p38α isoform and the upper band to p38γ, with the lower band being the most abundant^21^. Although we did not analyze the specific role of p38 isoforms, the use of a specific inhibitor for the p38α/β isoforms and the detection mainly of the lower band (Fig. 1) both suggest the involvement of the p38α isoform.

Activation of p38 is induced by most stress stimuli, including UV light, oxidative stress, and heat or osmotic shock, but also by exposure to cytokines, chemokines, hormones, and growth factors. One of the first reported p38 substrates was MK2. Active MK2 phosphorylates HSP27, resulting in actin filament remodeling and stimulation of cell migration^22^. Here, we show that HERC1 elimination increases HSP27 phosphorylation due to activation of p38, which results in an increase in cell migration. In addition, experiments in the presence of the inhibitor LY3009120 demonstrated that cell migration was dependent on RAF proteins that acted upstream of MKK3 and p38. Interestingly, the observed increase in cell migration due to HERC1 knockdown was completely rescued by inhibiting RAF activity or by knockdown of RAF isoforms, which implies that the observed effect of HERC1 on migration is mediated by RAF. Since HERC1 specifically regulates C-RAF isoform levels, it is highly likely that HERC1 regulation of migration is dependent on C-RAF. These observations agree with previous data showing that C-RAF regulates cell migration^9^. These authors correlated the defect in migration of C-RAF knockout (KO) cells with hyperactivity and incorrect localization of the Rho-associated kinase α (ROCKα) at the plasma membrane. Reintroduction of either wild-type or kinase-dead C-RAF in KO fibroblasts rescued the defect in migration. These latter observations suggest a kinase-independent function of C-RAF during migration^9^. Our experiments with LY3009120, a pan-RAF inhibitor of the three isoforms^15^, might indicate a kinase-dependent function. An explanation for these apparently contradictory conclusions might be that RAF proteins could act as heterodimers. Thus, reintroduction of kinase-dead C-RAF in KO fibroblasts could rescue the defect in migration, forming heterodimers with active A-ARF or B-RAF isoforms. Since RAF inhibitors such as LY3009120 or Sorafenib induce RAF dimerization^15^, another possibility could be that the C-RAF isoform is sequestered in inactive dimers (heterodimers or homodimers), preventing its interaction with ROCKα and inhibiting cell migration. In support of the involvement of Rho signaling in p38 activation during cell migration, it has been suggested that a Rho/ROCK/FAK/p38 signaling pathway mediates the stimulation of intestinal epithelial migration produced by repetitive deformation^23^, and that a Rho/ROCK/MKK3/p38 signaling pathway regulates sphingosine-1-phosphate-induced smooth muscle cell migration^24^. Other MAPK signaling pathways such as JNK or ERK also play crucial roles in cell migration. For example, ERK regulates cell movement by phosphorylating myosin light chain kinase, calpain, and focal-adhesion kinase^4,5^. Through C-RAF, HERC1 also regulates the ERK signaling pathway^8^. Thus, HERC1 regulates cell migration by modulating both ERK and p38 signaling pathways.

This study reflects the complex regulation of biological processes where ubiquitylation and phosphorylation mechanisms participate in an integrated manner to transmit signaling. Although more research is required to fully understand the functions of HERC1, its role in regulating cell migration (this study) and cell proliferation^8^ sheds light on why HERC1 mutations have been associated with serious pathologies, including tumorigenesis and neurological disorders^7^. In this context, FDA-approved RAF inhibitors are potential candidates for use as therapeutic drugs in these pathologies.

## Material and Methods

### Reagents

We used the following reagents: anti-p-p38 (Cell Signaling); anti-p-HSP27 (Enzo Life Sciences); anti-p38, anti-HSP27, anti-α-tubulin and anti-β-actin (Santa Cruz Biotechnology); anti-MKK3 and anti-MKK6 (Proteintech Europe); anti-p44/42 MAPK (ERK1/2) (Cell Signaling); anti-p-ERK1/2 (Sigma-Aldrich); anti-HERC1 (Bvg6, 411, 363, 410)^25^; anti-C-RAF and anti-clathrin heavy chain (BD Biosciences); anti-A-RAF(A-5) and anti-B-RAF (F-7) (Santa Cruz Biotechnology); ubiquitylated proteins (clone FK2) (Biomol); anti-c-myc (clone 9E10) (Roche); anti-Flag M2 (Sigma-Aldrich); DAPI (Sigma-Aldrich); phalloidin-Alexa 647 (BioProbes); conjugated secondary antibodies to Alexa-Fluor 488 and horseradish peroxidase-conjugated secondary antibodies (Invitrogen); Immobilon-P PVDF transfer membrane (Millipore Corporation); SB203580 (Selleckchem); U0126 (Calbiochem); LY3009120 (Selleckchem); Sorafenib (Santa Cruz Biotech.); MG132 (Merck Millipore); anti-Flag M2 affinity gel (Sigma-Aldrich); Luminescent β-galactosidase detection Kit II (Clontech Laboratories); and Luciferase assay system (Promega).

### Plasmids and siRNAs

pRc/RSV-Flag-MKK3 and pcDNA3 constructs were purchased from Addgene and Invitrogen, respectively. shRNA and siRNA were previously described^8^. Briefly, human HERC1 shRNA (TRCN0000235499) and vector pLKO.1-Puro were from Sigma-Aldrich. siRNAs were purchased from GenePharma: non-targeting (NT, UAGCGACUAAACACAUCAA), HERC1 (Q1, CGGCAUGGAUGAACAAAUU and Q4, GGGCAGAACUUCGUUUAGA), A-RAF (AACAACATCTTCCTACATGAG), B-RAF (AAAGAATTGGATCTGGATCAT) and C-RAF (UAGUUCAGCAGUUUGGCUATT).

### Transfections and cell culture

U2OS, HEK-293T and MEFs cells were cultured at 37 °C in DMEM (Dulbecco’s Modified Eagle’s Medium, Gibco) with 10% fetal bovine serum. Transfection of cells was performed as previously described^8^ using Lipofectamine LTX (Invitrogen) for plasmids and calcium phosphate for siRNAs. Specific inhibitors were used at a final concentration of 10 μM (U0126, SB203580, Sorafenib and LY3009120 for 1 hour, and MG132 for 6 hours). Preparation and infection of lentiviral particles were carried out according to the manufacturer’s instructions (Sigma-Aldrich).

### Lysates and immunoblotting

Lysates were prepared with NP40 buffer as previously described^8^ and centrifuged at 13,000 x g at 4 °C for 10 min. Supernatants were analyzed by electrophoresis and immunoblotting using the Tris-acetate PAGE system^26^. Band intensities were analyzed using a gel documentation system (LAS-3000 Fujifilm). Protein levels were normalized and expressed as a percentage of controls. The levels of phosphorylated proteins (p-p38, p-ERK1/2, and p-HSP27) were normalized by their total amount. Raw data are shown in supplementary information.

### Immunoprecipitation and pull-downs

For Flag pull-downs, the above supernatants were incubated at 4 °C with 5 μL of anti-Flag M2 affinity gel for 2 hours. Next, they were centrifugated and pull-downs were washed with NP40 buffer (five times) and analyzed by electrophoresis and immunoblotting as indicated above. For immunoprecipitation (IP), supernatants (input) were incubated at 4 °C with anti-HERC1 polyclonal antibodies (Bvg6, 411, 363, 410) or with pre-immune serum (PI) for 2 hours. Next, protein A-Sepharose was added for 1 hour. Beads were pelleted by centrifugation at 2,500 g, washed with NP40 buffer (four times), and analyzed as indicated above.

### Confocal microscopy

U2OS cells grown on glass coverslips were fixed at room temperature for 20 min with 4% paraformaldehyde. Cells were permeabilized for 20 minutes with 0.05% saponin in PBS containing 0.5% bovine serum albumin. The primary antibody, anti-p-p38 (1:200), was incubated at 37 °C for 1 hour. After washing, Alexa-Fluor 488 secondary antibody (1:500) was incubated at 37 °C for 45 minutes. Incubation with phalloidin-Alexa 647 (100 ng/mL) for 20 minutes at room temperature was used to detect F-actin. Nuclei were stained with DAPI (1 μg/mL). Images were acquired using a confocal laser scanning microscope (LSM 880 spectral, Carl Zeiss Microscopy GmbH, Jena, Germany).

### Reverse transcriptase quantitative PCR analysis (RT-qPCR)

Total RNA was isolated from U2OS cells using Trisure reagent following the manufacturer’s protocol (Bioline). Total RNA (2 μg) was reverse-transcribed using the high capacity cDNA Reverse Transcription kit (Applied Biosystems). PCR amplification reactions were performed with the ABI Prism 7900 HT Fast Real-Time PCR System. Applied Biosystems’ TaqMan Gene Expression Assays were used to quantify gene expression of MKK3 (Hs00177127_m1) and the housekeeping gene TBP (HS99999910_m1), which was used to normalize. Data from PCR were acquired and analyzed using Sequence Detector software (Applied Biosystems, SDS version 2.3).

### Wound healing assay

Following 48 h of siRNA transfection, U2OS cells (3 × 10^5^/well) were seeded in 6-well plates. When the cells reached 100% confluency, the cells were non-treated or treated with 10 μM of SB203580 or LY3009120 for 1 hour. Subsequently, a cell-free area was created (linear wound). The scratch closure was monitored and documented after 0, 3, 6, and 12 hours, using a Leica DM IRB Inverted Research Microscopy (Leica Microsystems Wetzlar GmbH). Similar results were obtained when cells at 100% confluency were pretreated with 10 μg/mL mitomycin C for 2 h. Wound area was determined using ImageJ software.

### Statistical analysis

Results were analyzed using GraphPad Prism 5 software. Data are expressed as mean ± S.E.M. Differences between groups were analyzed using the Student’s *t*-test or one-way analysis of variance (ANOVA) with Bonferroni’s multiple comparison test. Statistical significance was set at p < 0.05.

## Supplementary information

Raw data.
