# Supplementary material for: The ubiquitin ligase HERC1 regulates cell migration via RAF-dependent regulation of MKK3/p38 signaling
Source: Sci Rep. 2020 Jan 21;10:824. doi: 10.1038/s41598-020-57756-7 (PMC6972752; doi:10.1038/s41598-020-57756-7)
Supplement: Supplementary file 1 — Raw data. [file 41598_2020_57756_MOESM1_ESM.pdf]

The ubiquitin ligase HERC1 regulates cell migration via RAF-dependent regulation of MKK3/p38 signaling

Leonardo Pedrazza[1,*], Taiane Schneider[1,*], Ramon Bartrons[1], Francesc Ventura[1] and Jose Luis Rosa[1,#]

[1] Departament de Ciències Fisiològiques, Institut d'Investigació de Bellvitge (IDIBELL), Universitat de Barcelona, L'Hospitalet de Llobregat, Barcelona, Spain.

[*] : These authors contributed equally to this work.

[#] : Corresponding author: joseluisrosa@ub.edu

ORCID iD for Jose Luis Rosa: 0000-0002-6161-5688

Fig. 1

A

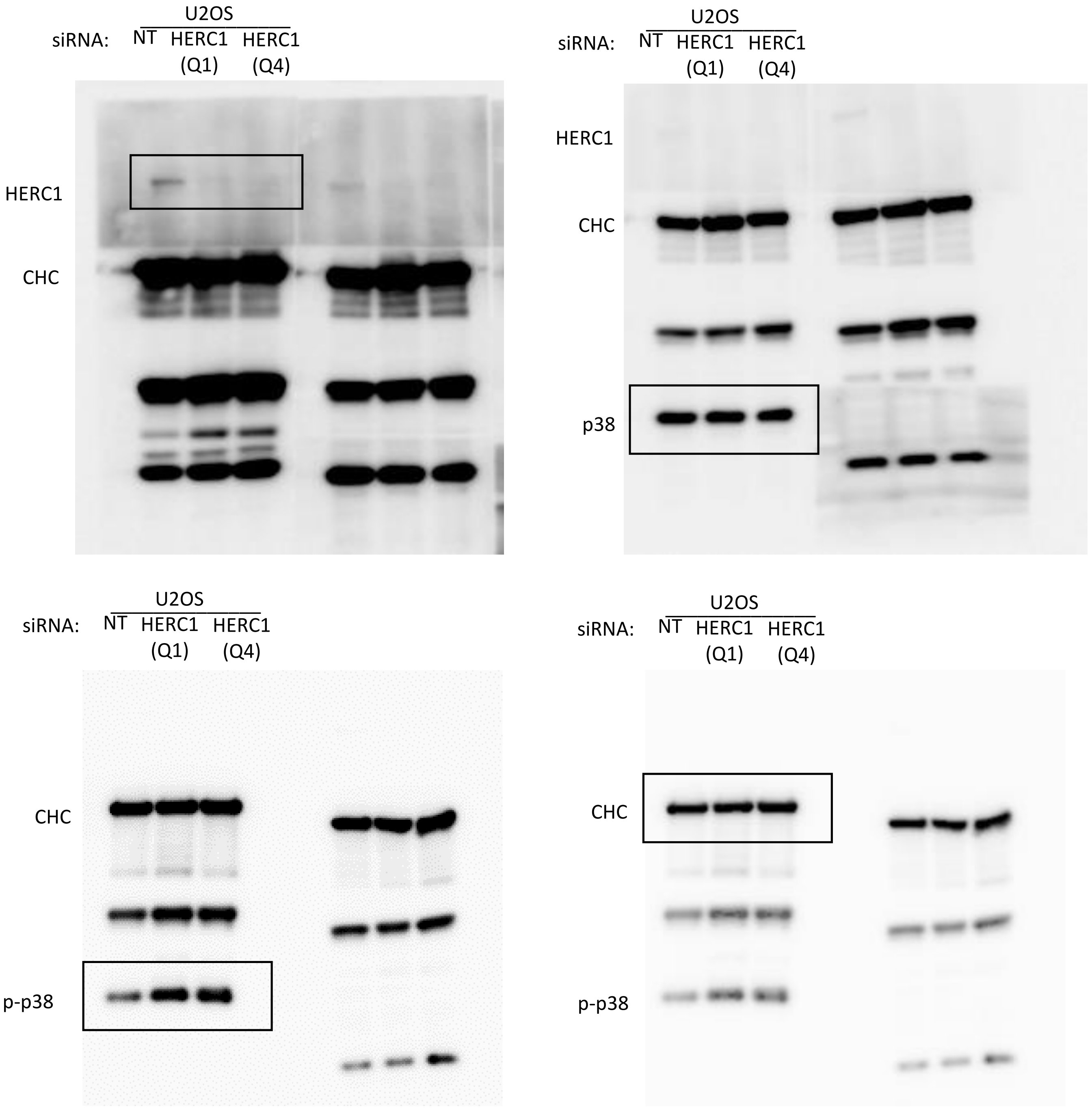

Fig. 1

A

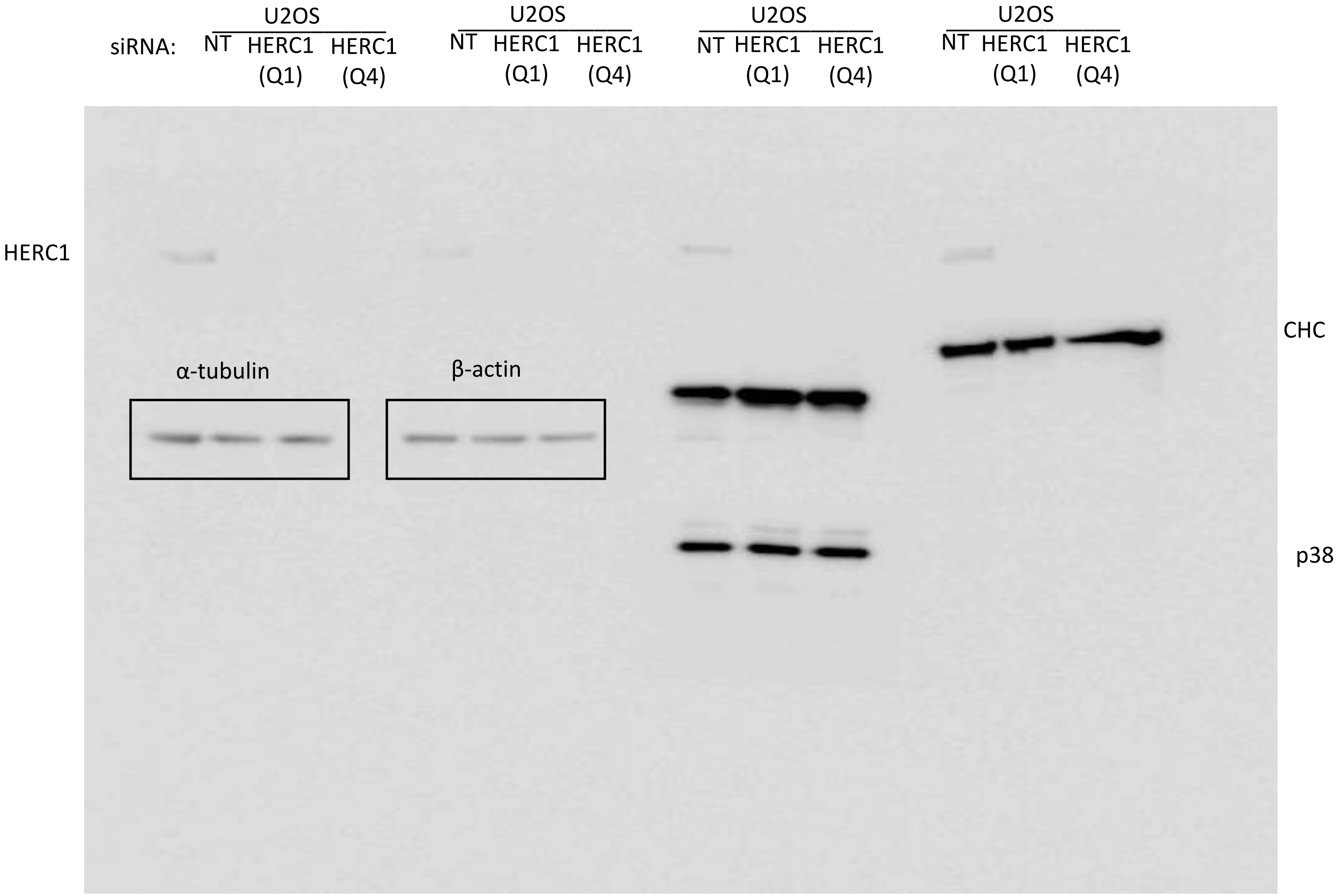

U2OS
siRNA: NT HERC1 (Q1) HERC1 (Q4)
U2OS
NT HERC1 (Q1) HERC1 (Q4)
U2OS
NT HERC1 (Q1) HERC1 (Q4)
U2OS
NT HERC1 (Q1) HERC1 (Q4)
HERC1
α-tubulin
β-actin
CHC
p38

Fig. 1

B

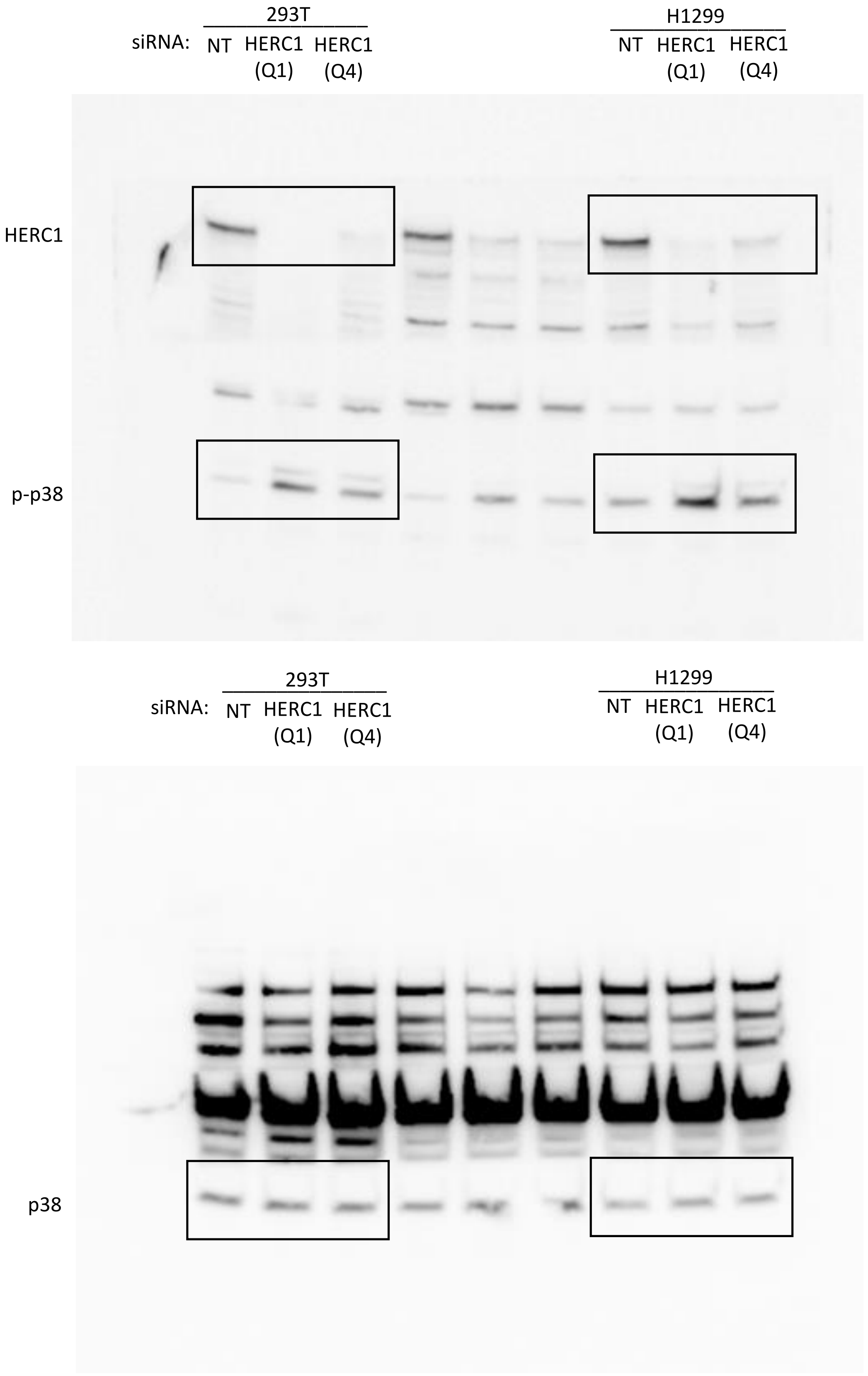
293T
siRNA:
NT
HERC1 (Q1)
HERC1 (Q4)
H1299
NT
HERC1 (Q1)
HERC1 (Q4)
HERC1
p-p38
293T
siRNA:
NT
HERC1 (Q1)
HERC1 (Q4)
H1299
NT
HERC1 (Q1)
HERC1 (Q4)
p38

Fig. 1

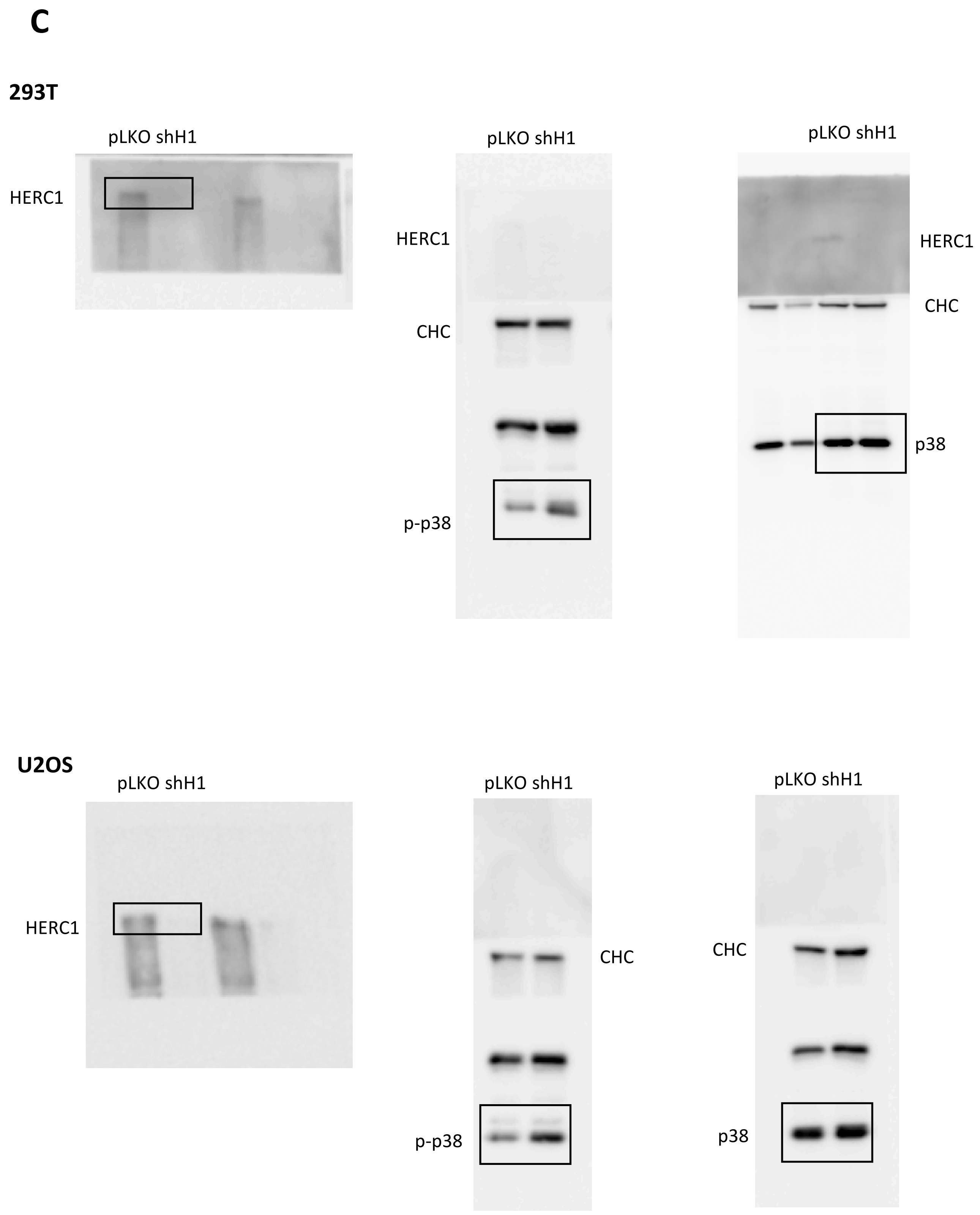
C
293T
pLKO shH1
HERC1
pLKO shH1
HERC1
CHC
p-p38
pLKO shH1
HERC1
CHC
p38
U2OS
pLKO shH1
HERC1
pLKO shH1
CHC
p-p38
pLKO shH1
CHC
p38

Fig. 1

D

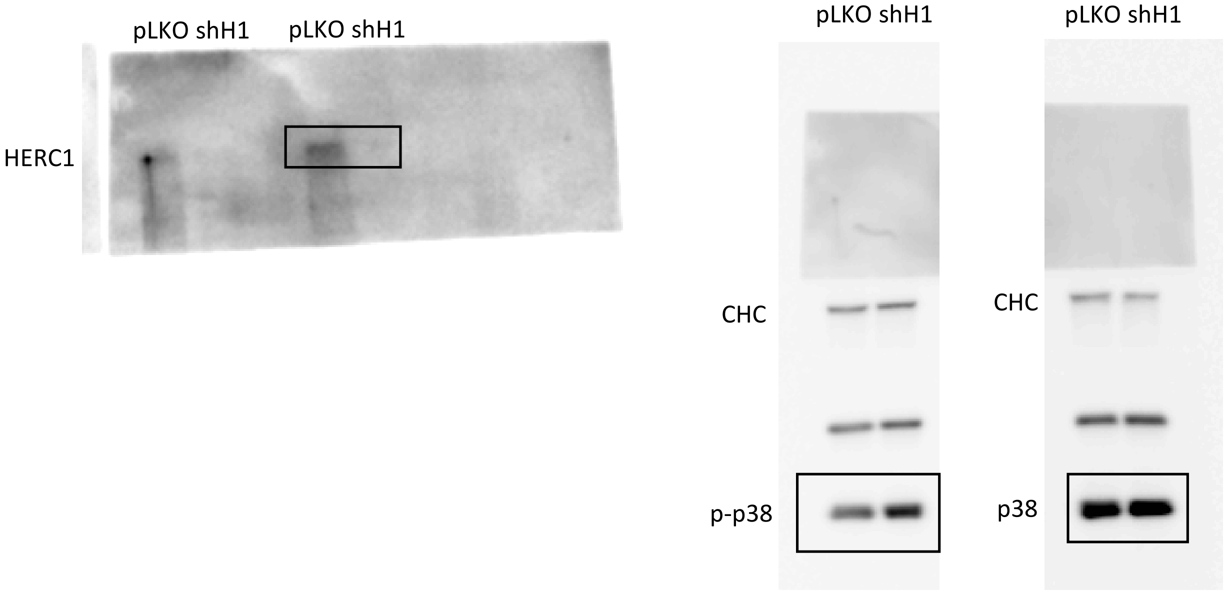
pLKO shH1
pLKO shH1
HERC1
pLKO shH1
pLKO shH1
CHC
p-p38
CHC
p38

Fig. 1

E

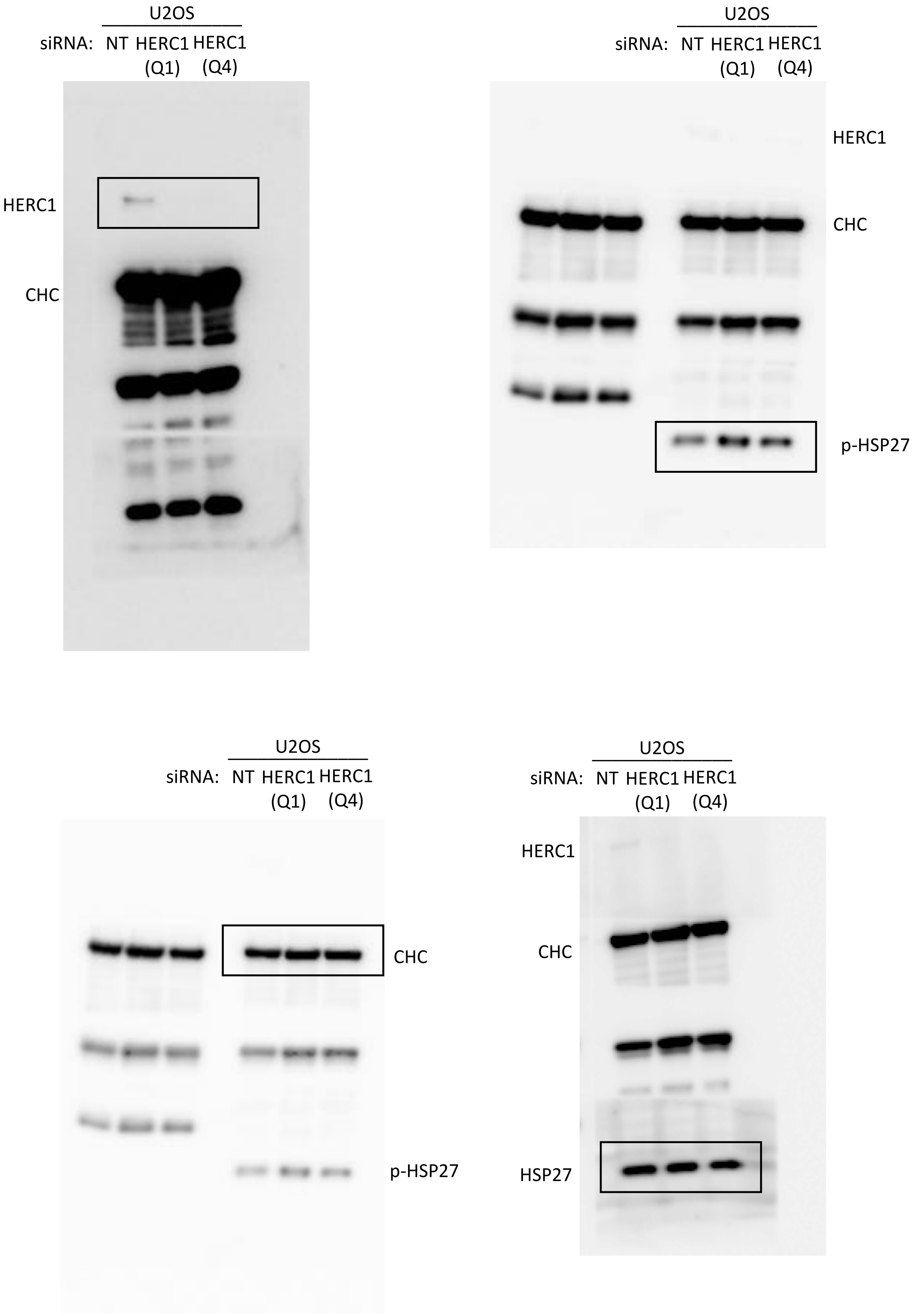
U2OS
siRNA: NT HERC1 (Q1) HERC1 (Q4)
HERC1
CHC
U2OS
siRNA: NT HERC1 (Q1) HERC1 (Q4)
HERC1
CHC
p-HSP27
U2OS
siRNA: NT HERC1 (Q1) HERC1 (Q4)
CHC
p-HSP27
U2OS
siRNA: NT HERC1 (Q1) HERC1 (Q4)
HERC1
CHC
HSP27

Fig. 1

F

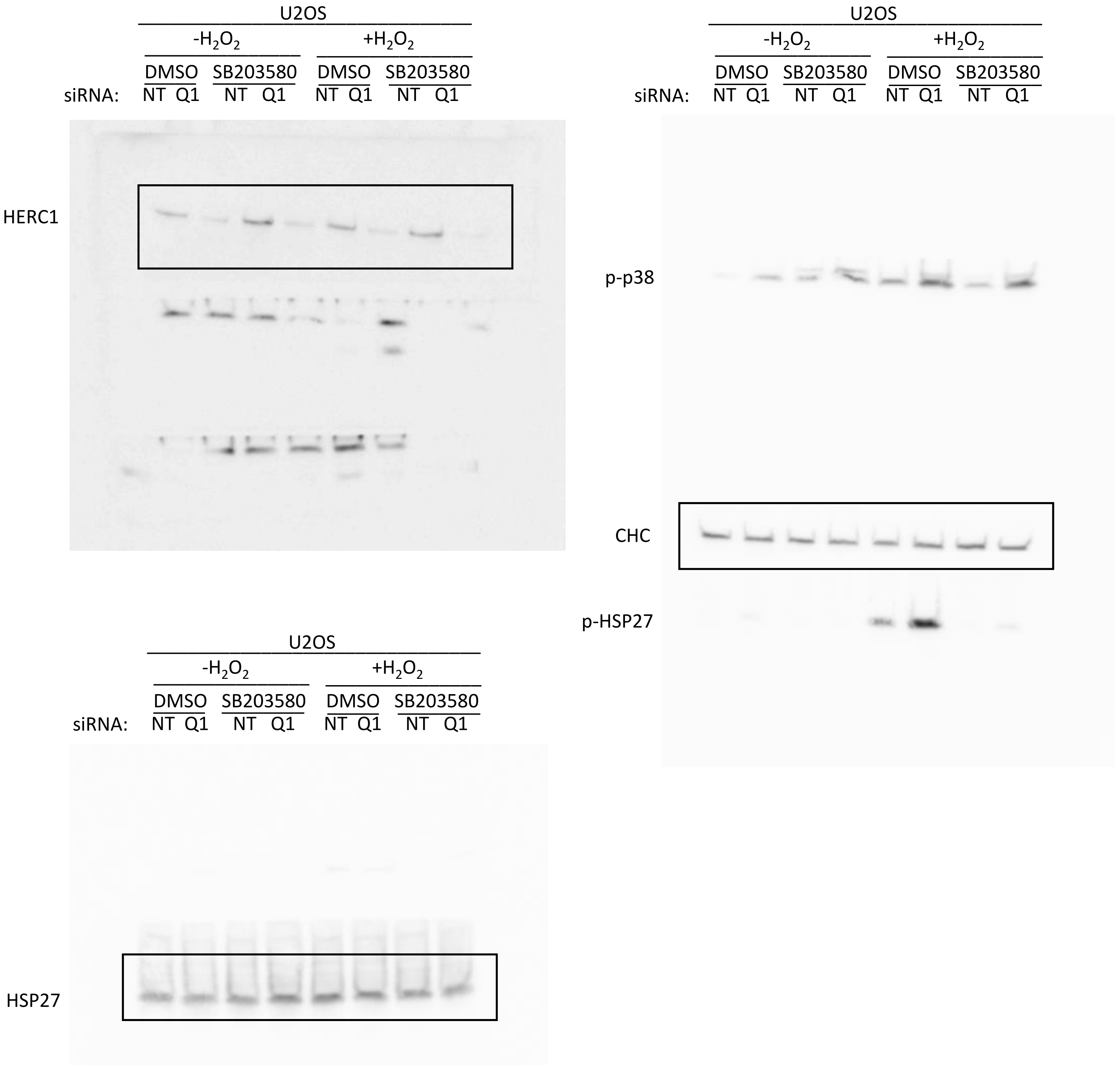

U2OS
-H2O2
+H2O2
DMSO
SB203580
siRA:
NT
Q1
HERC1
p-p38
CHC
p-HSP27
HSP27

Fig. 1

F

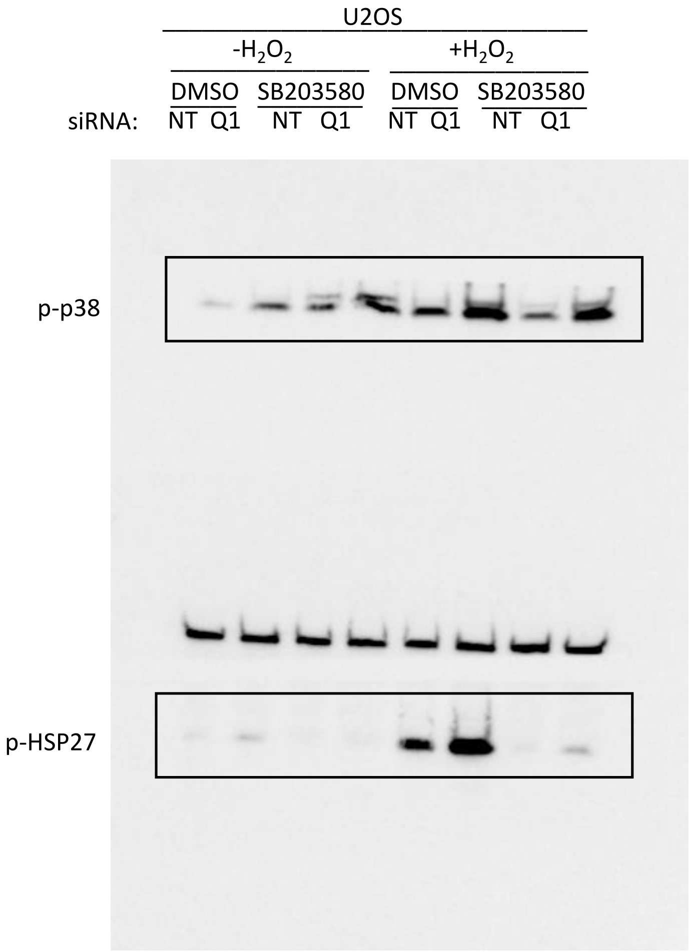
U2OS
$-H_2O_2$
$+H_2O_2$
DMSO
SB203580
DMSO
SB203580
siRNA:
NT Q1 NT Q1 NT Q1 NT Q1
p-p38
p-HSP27

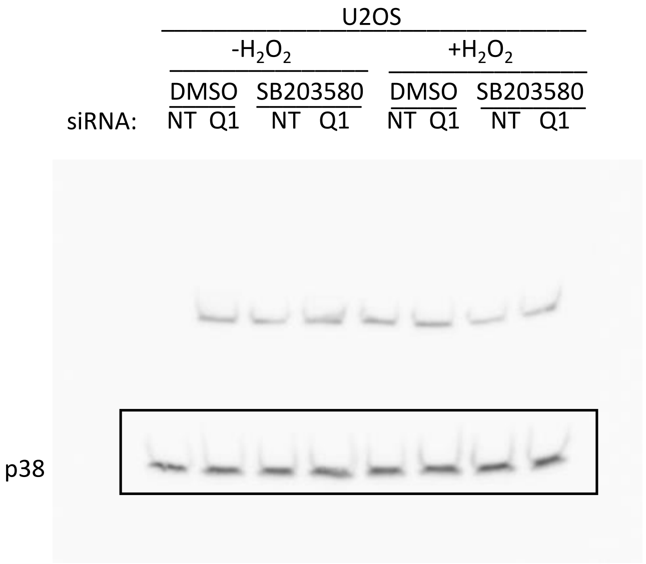
U2OS
$-H_2O_2$
$+H_2O_2$
DMSO
SB203580
DMSO
SB203580
siRNA:
NT Q1 NT Q1 NT Q1 NT Q1
p38

Fig. 2

A

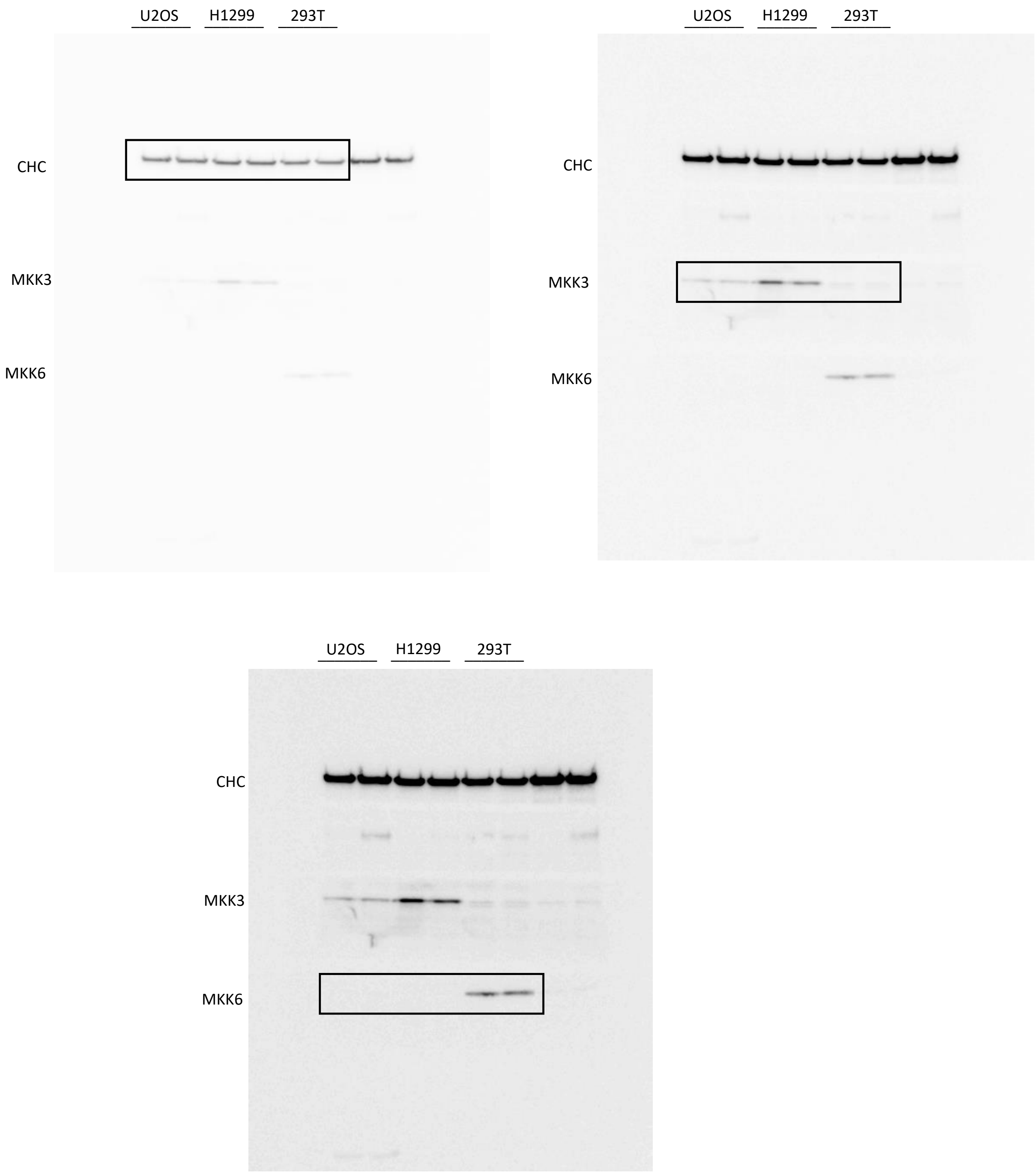
U2OS
H1299
293T
CHC
MKK3
MKK6
U2OS
H1299
293T
CHC
MKK3
MKK6
U2OS
H1299
293T
CHC
MKK3
MKK6

Fig. 2

B

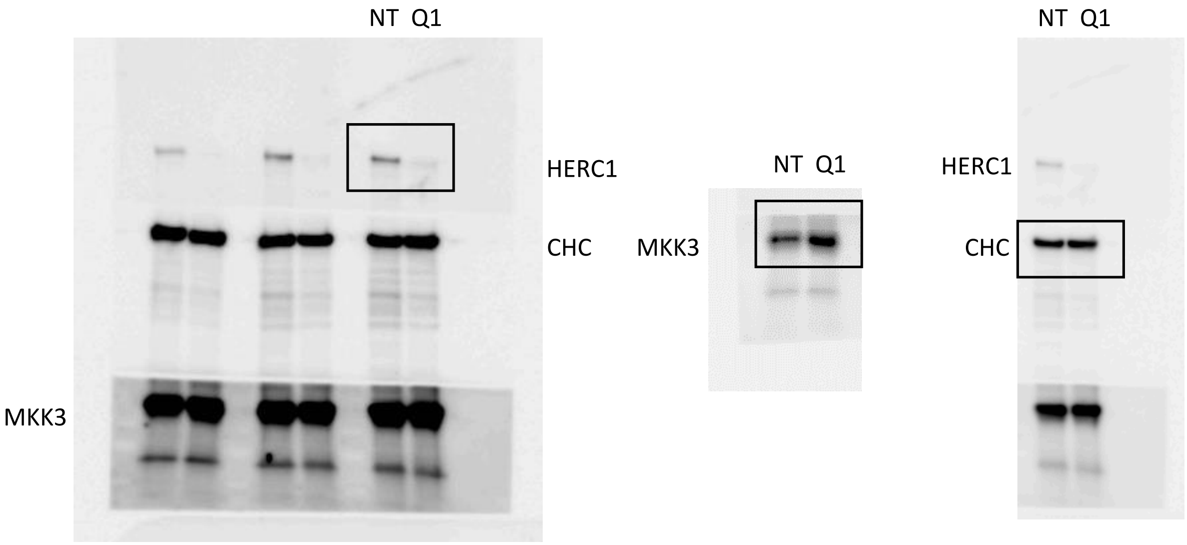

Fig. 2

C

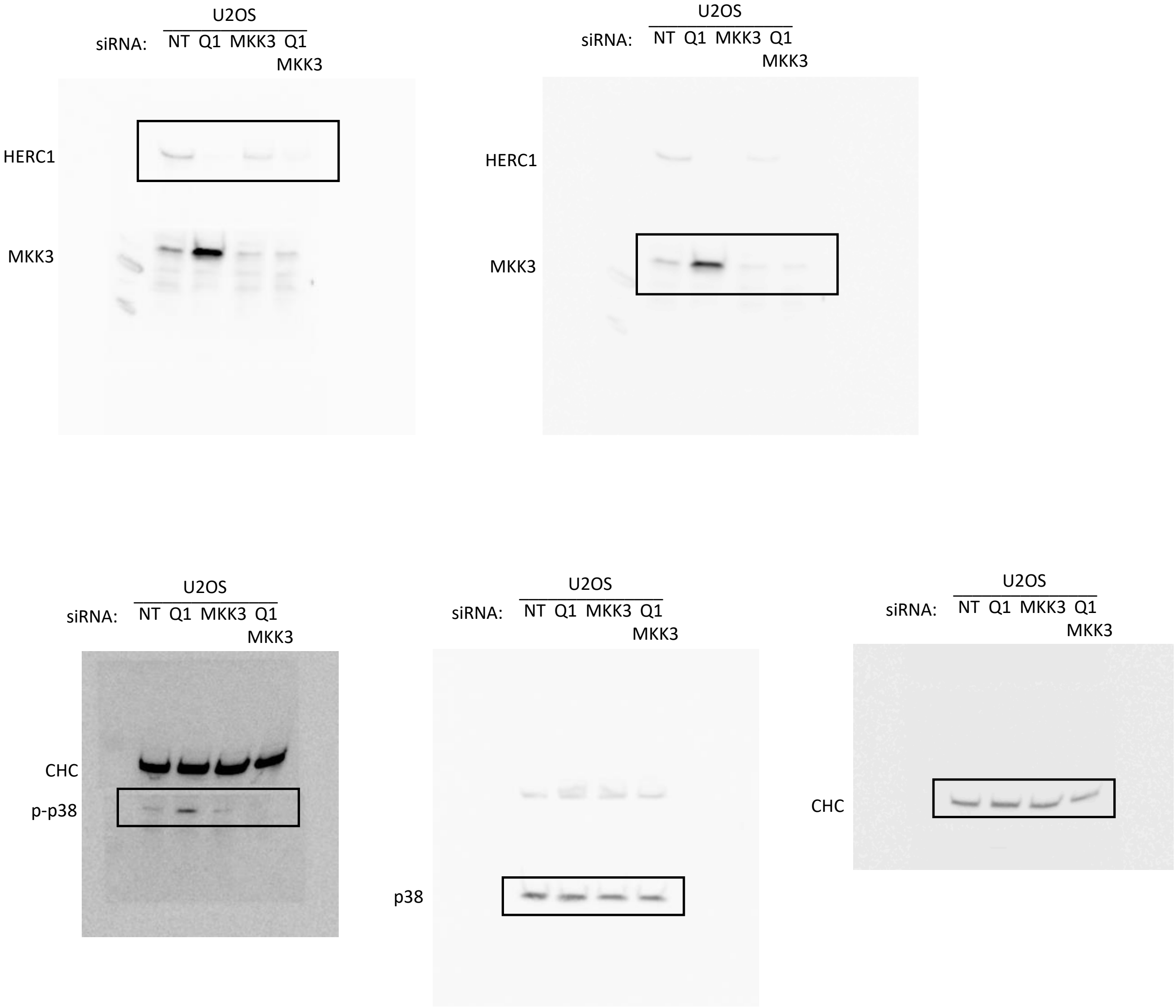

U2OS
siRNA: NT Q1 MKK3 Q1 MKK3
HERC1
MKK3
U2OS
siRNA: NT Q1 MKK3 Q1 MKK3
HERC1
MKK3
U2OS
siRNA: NT Q1 MKK3 Q1 MKK3
CHC
p-p38
U2OS
siRNA: NT Q1 MKK3 Q1 MKK3
p38
U2OS
siRNA: NT Q1 MKK3 Q1 MKK3
CHC

Fig. 2

D

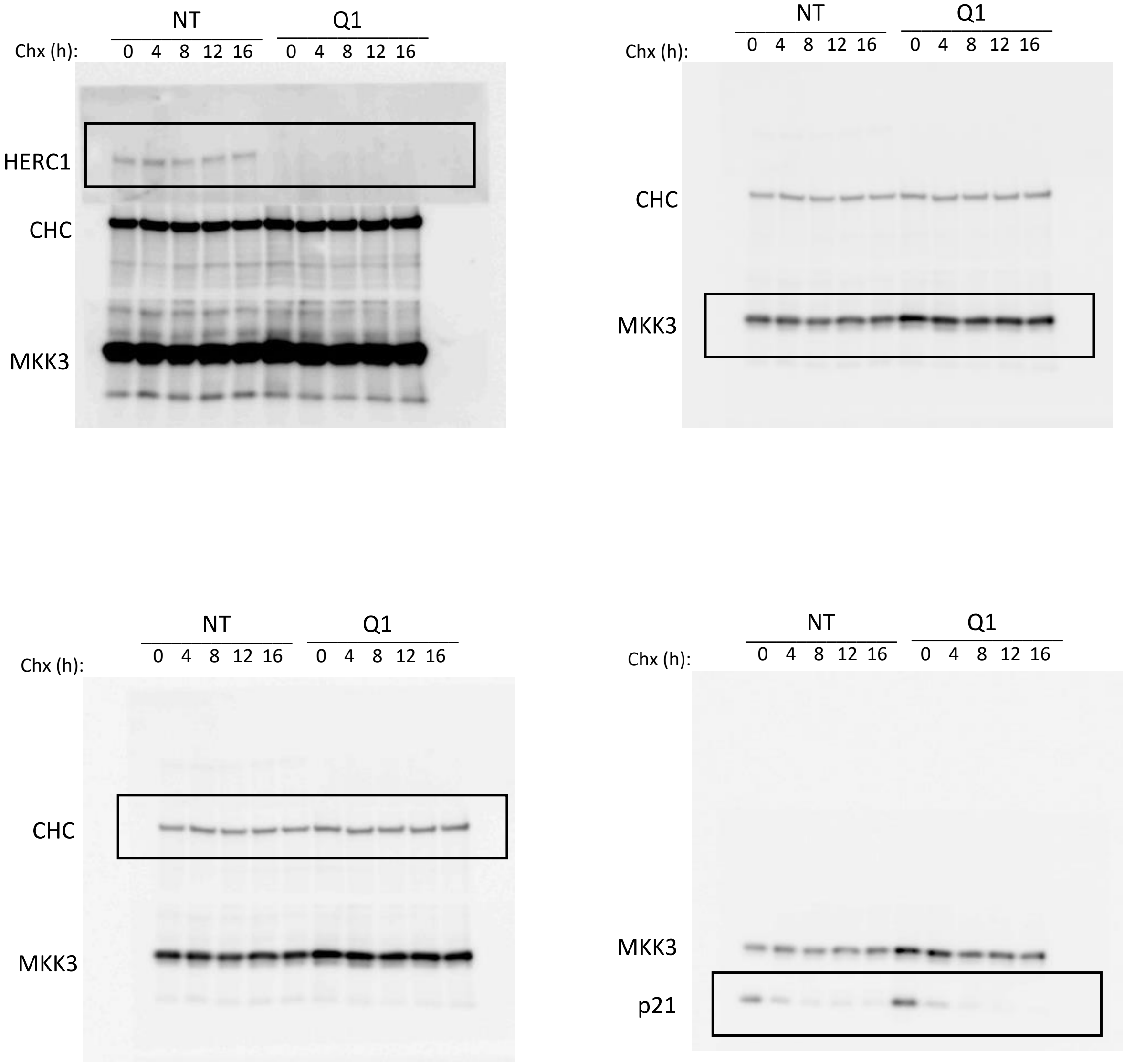

NT
Q1
Chx (h):
0 4 8 12 16 0 4 8 12 16
HERC1
CHC
MKK3
NT
Q1
Chx (h):
0 4 8 12 16 0 4 8 12 16
CHC
MKK3
NT
Q1
Chx (h):
0 4 8 12 16 0 4 8 12 16
CHC
MKK3
NT
Q1
Chx (h):
0 4 8 12 16 0 4 8 12 16
MKK3
p21

Fig. 2

F

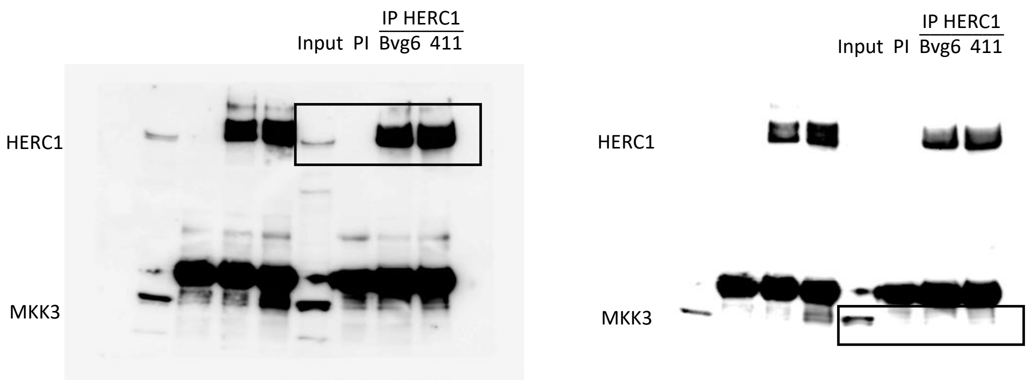
IP HERC1
Input PI Bvg6 411
HERC1
MKK3
IP HERC1
Input PI Bvg6 411
HERC1
MKK3

Fig. 2

G

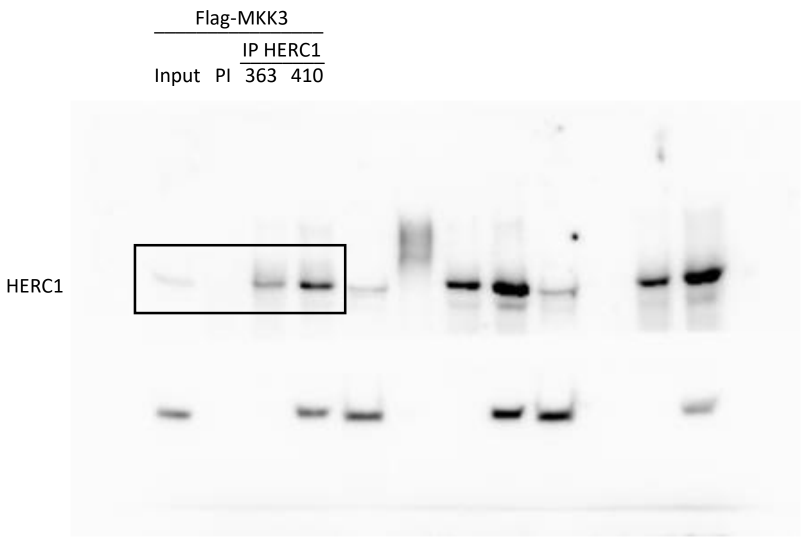

Flag-MKK3
IP HERC1
Input PI 363 410
HERC1

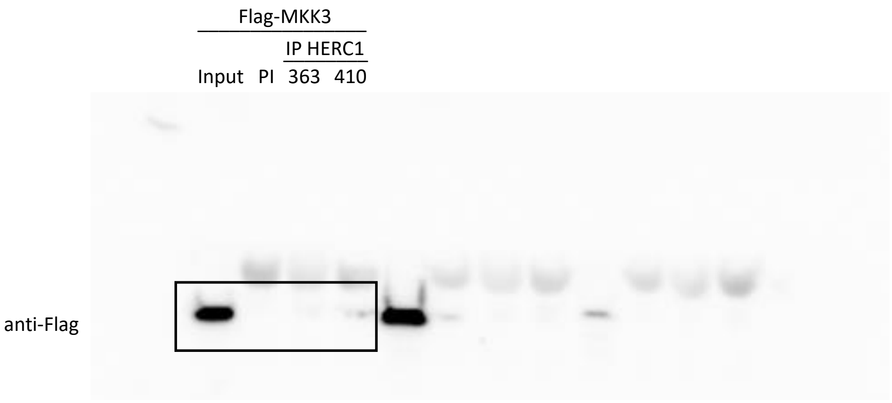

Flag-MKK3
IP HERC1
Input PI 363 410
anti-Flag

Fig. 2

H

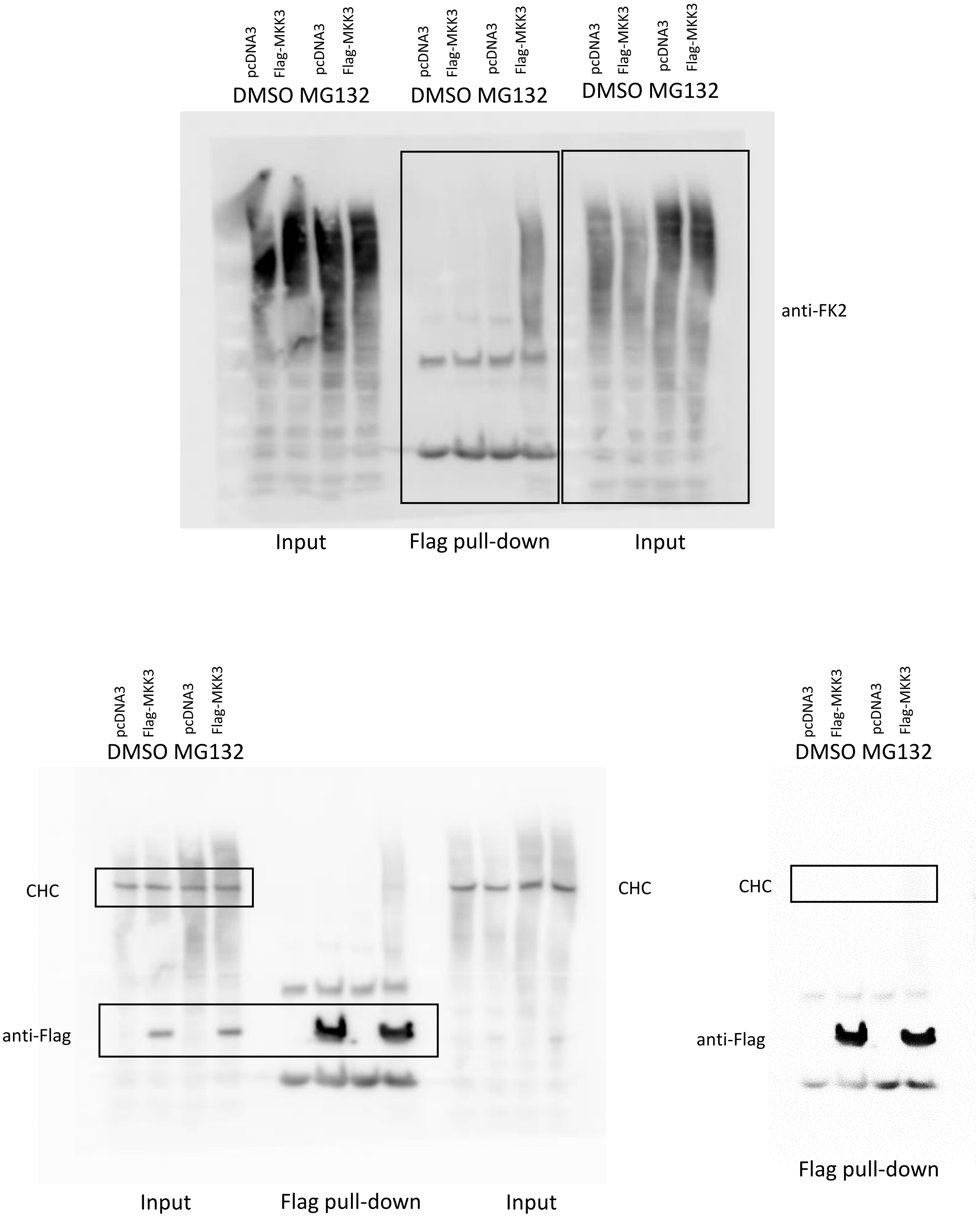
pcDNA3
Flag-MKK3
DMSO MG132
anti-FK2
Input
Flag pull-down
Input
CHC
anti-Flag
CHC
Flag pull-down

Fig. 2

I

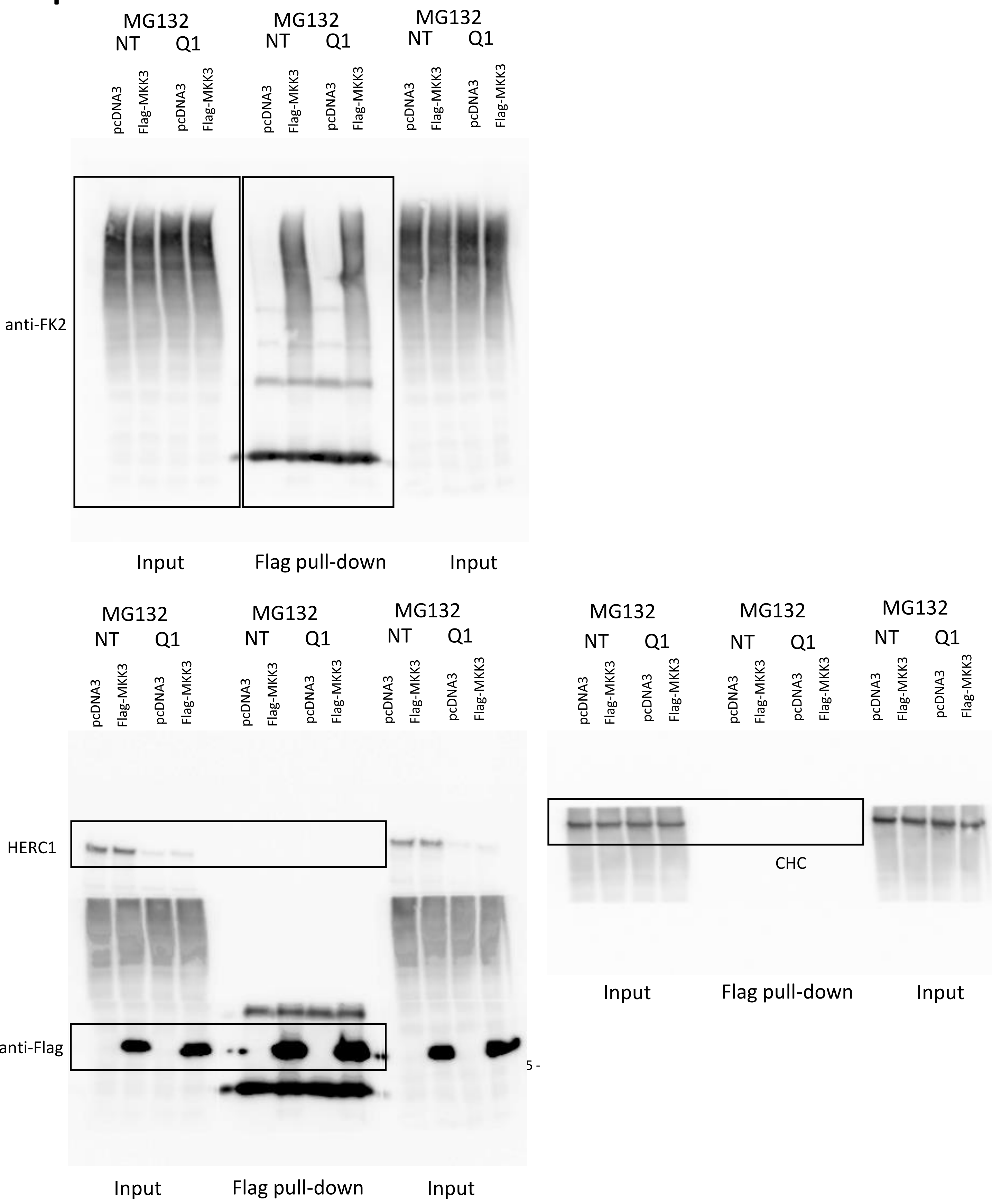
MG132
NT Q1
pcDNA3
Flag-MKK3
anti-FK2
Input
Flag pull-down
Input
HERC1
anti-Flag
CHC
Input
Flag pull-down
Input

Fig. 3

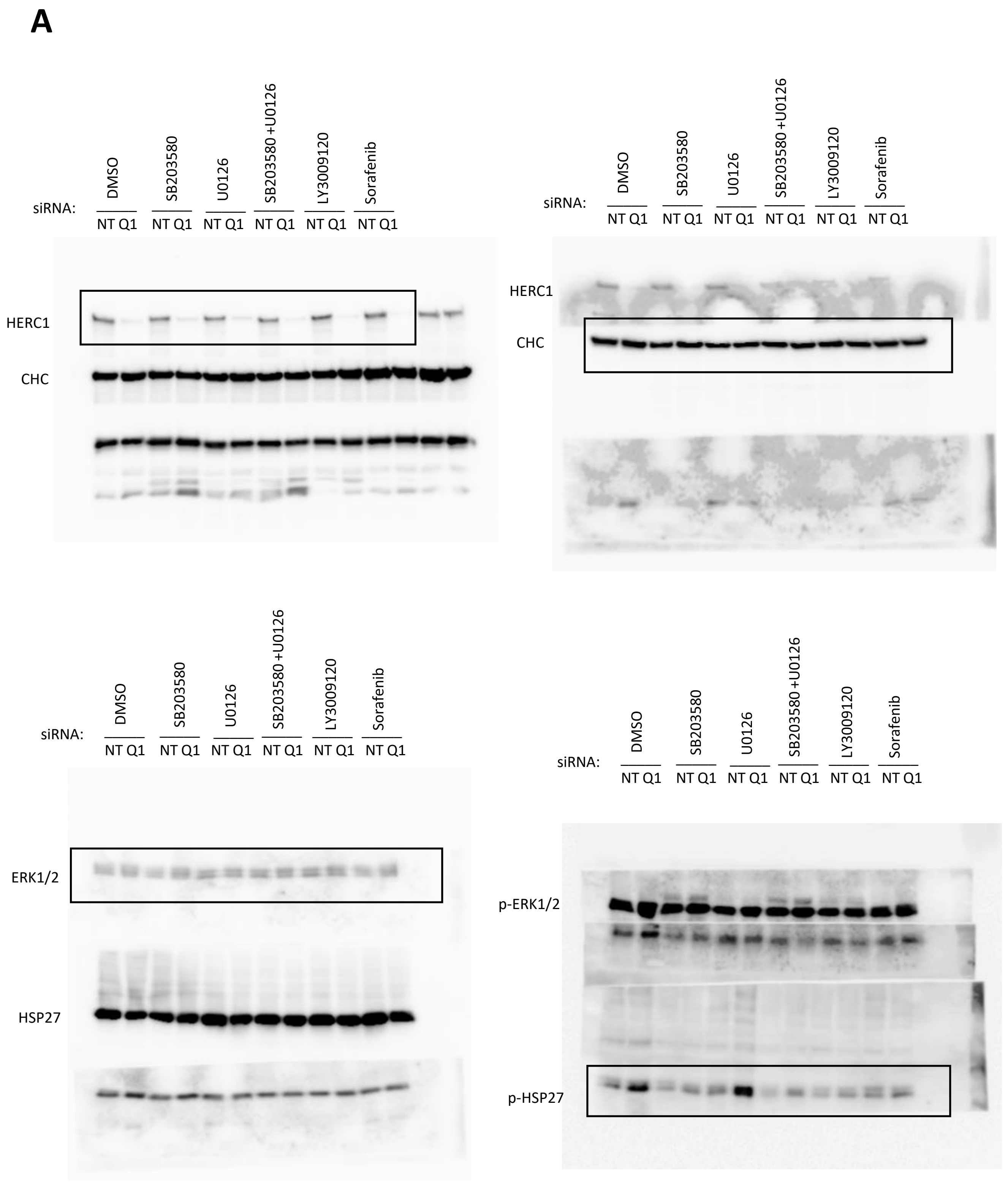
A
siRNA:
DMSO
SB203580
U0126
SB203580+U0126
LY3009120
Sorafenib
NT Q1 NT Q1 NT Q1 NT Q1 NT Q1 NT Q1
HERC1
CHC
ERK1/2
HSP27
p-ERK1/2
p-HSP27

Fig. 3

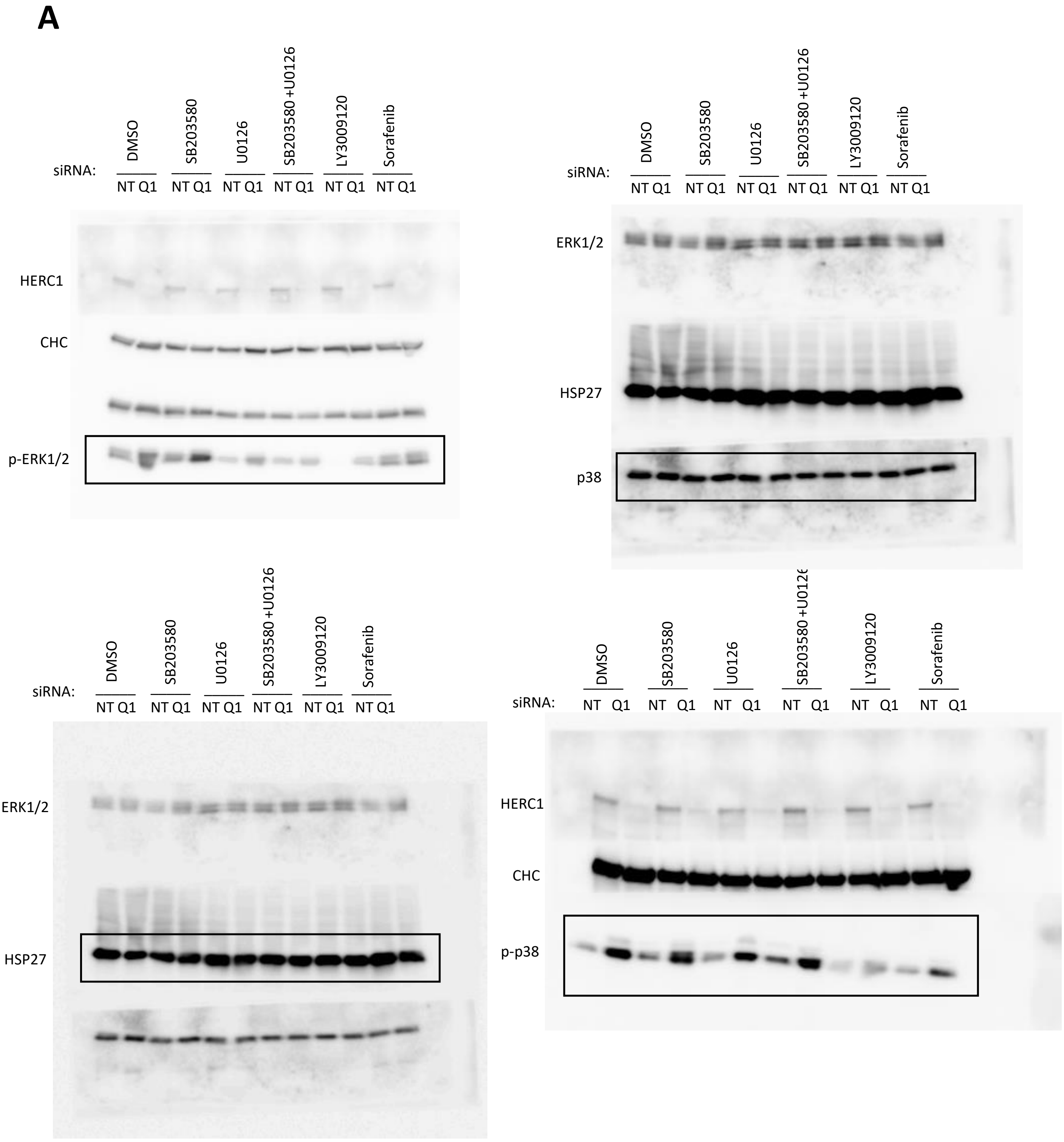
A
siRNA:
DMSO
SB203580
U0126
SB203580 +U0126
LY3009120
Sorafenib
NT Q1
HERC1
CHC
p-ERK1/2
ERK1/2
HSP27
p38
p-p38

Fig. 3

B

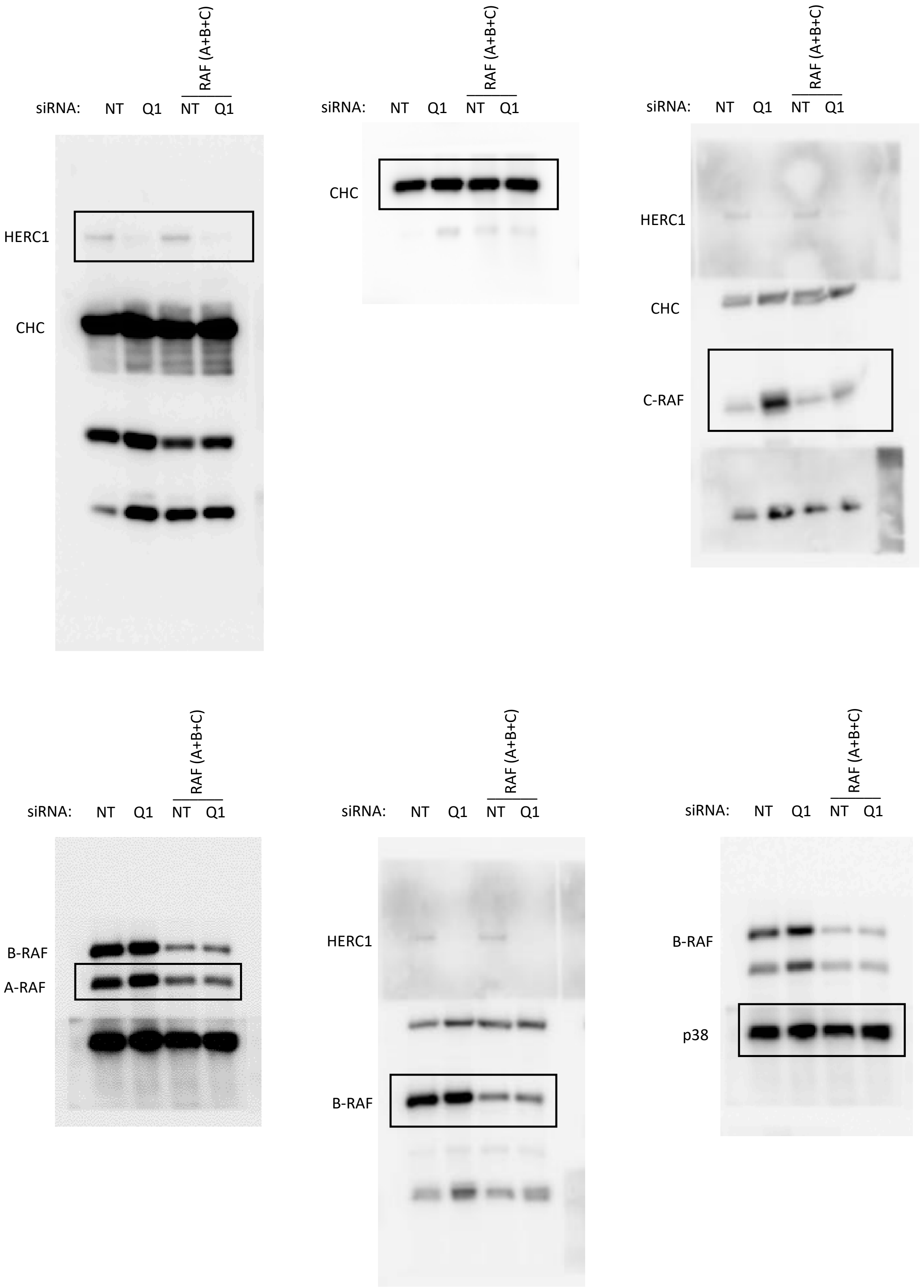

Fig. 3

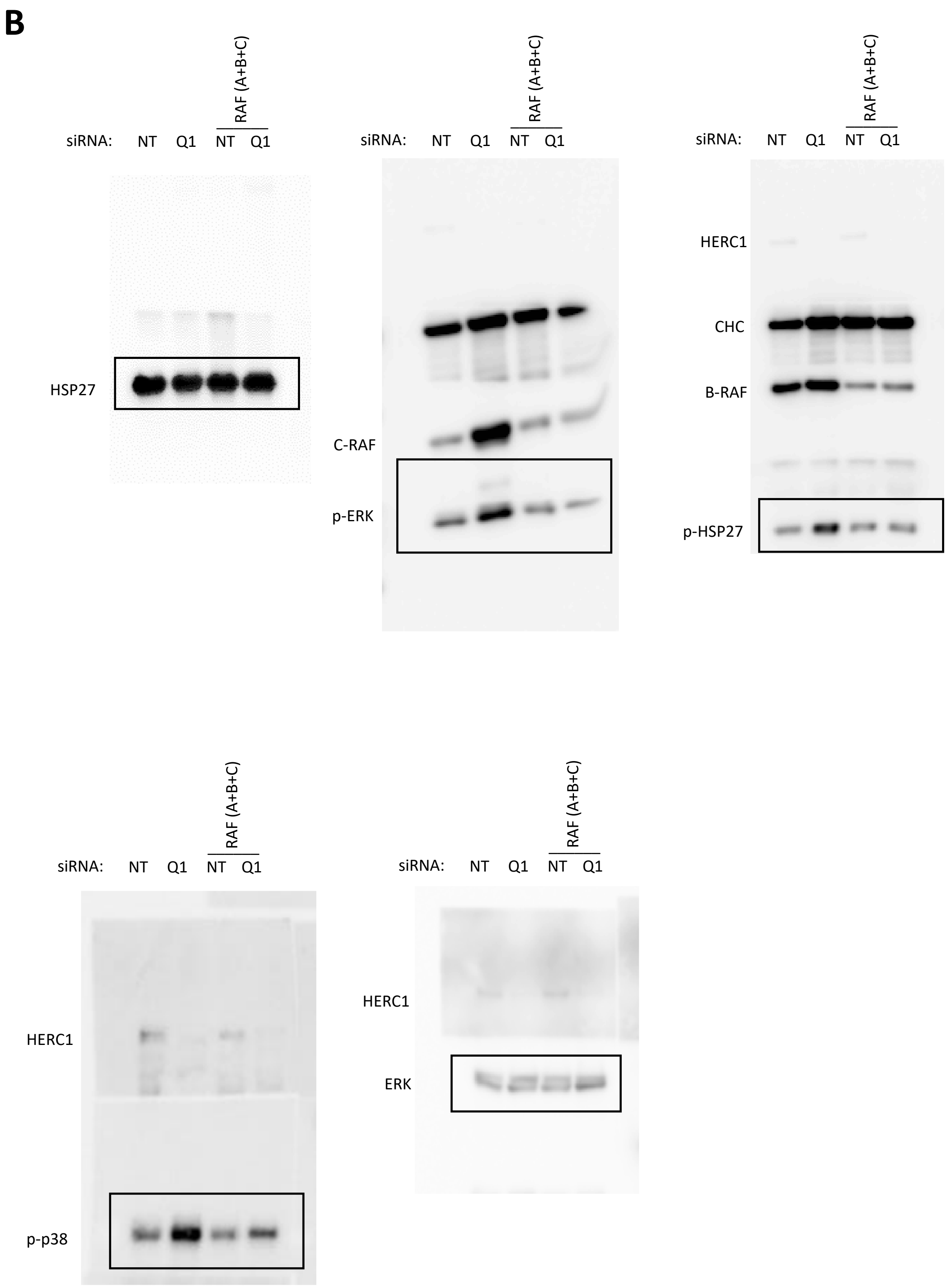
B
siRNA: NT Q1 NT Q1
RAF (A+B+C)
HSP27
siRNA: NT Q1 NT Q1
RAF (A+B+C)
C-RAF
p-ERK
siRNA: NT Q1 NT Q1
RAF (A+B+C)
HERC1
CHC
B-RAF
p-HSP27
siRNA: NT Q1 NT Q1
RAF (A+B+C)
HERC1
p-p38
siRNA: NT Q1 NT Q1
RAF (A+B+C)
HERC1
ERK

Fig. 3

C

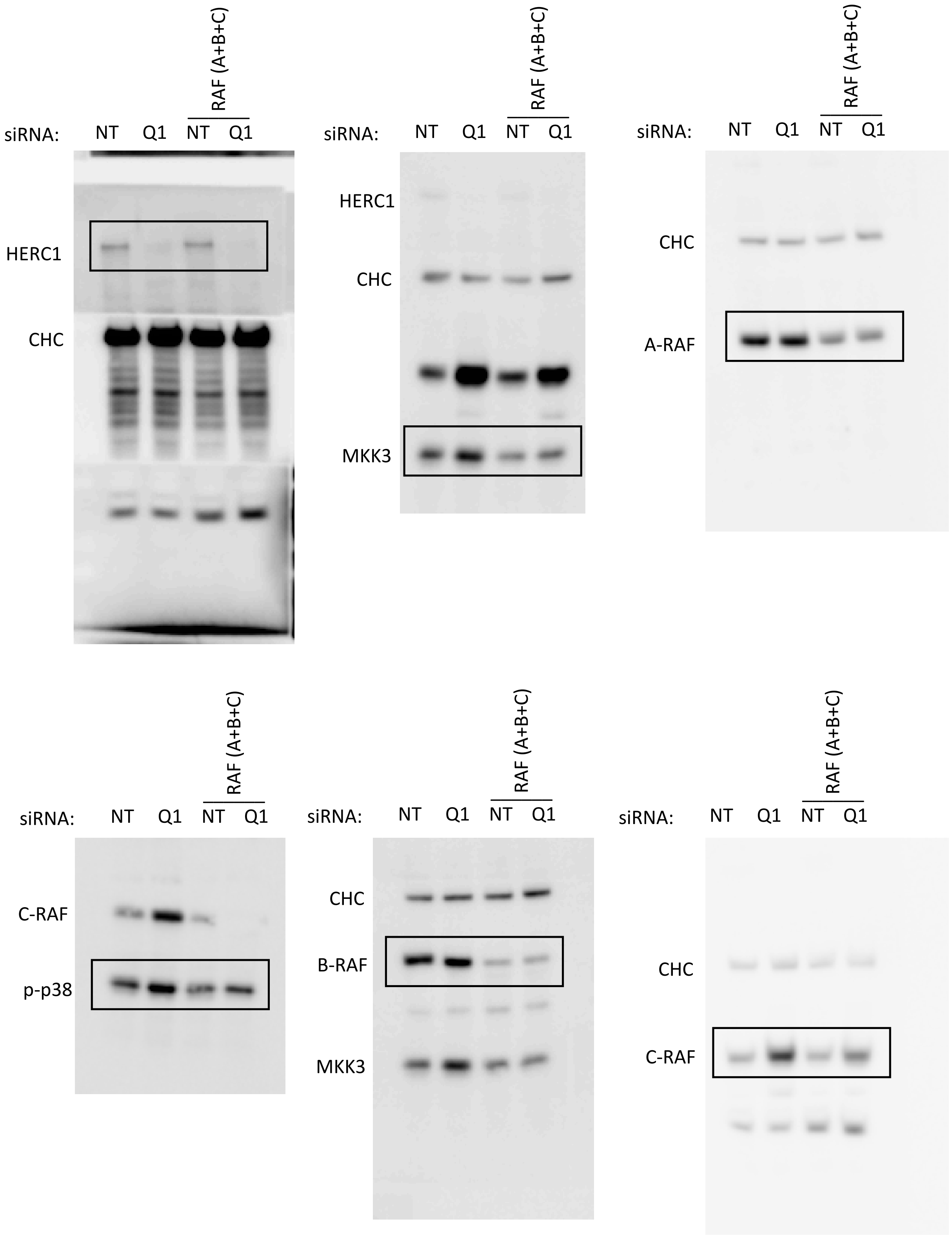

Fig. 3

C

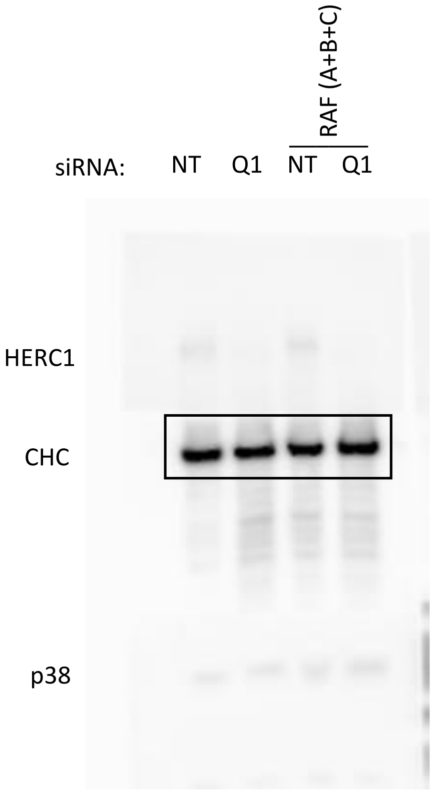

RAF (A+B+C)
siRNA:
NT
Q1
NT
Q1
HERC1
CHC
p38

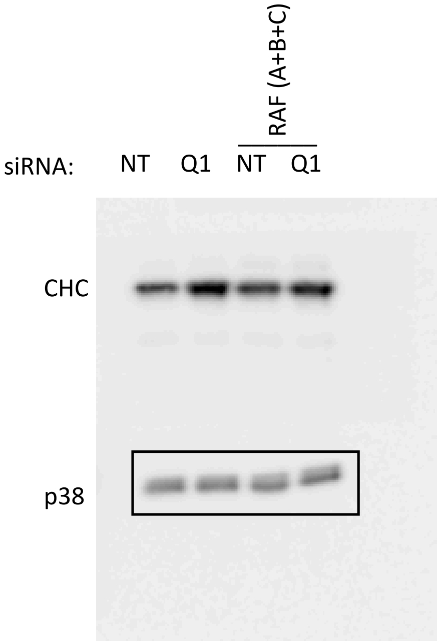

RAF (A+B+C)
siRNA:
NT
Q1
NT
Q1
CHC
p38

Fig. 4

A

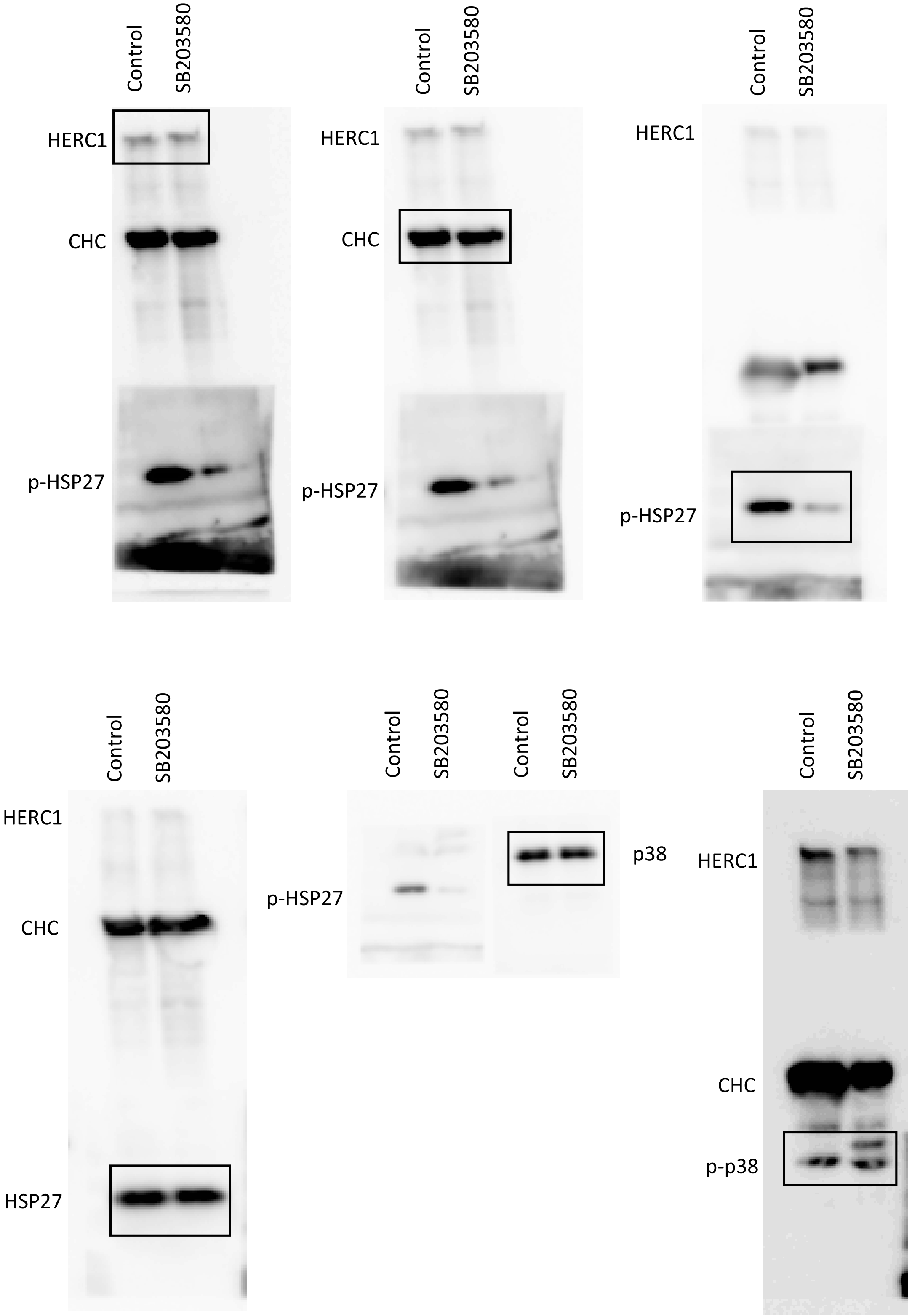
Control
SB203580
HERC1
CHC
p-HSP27
Control
SB203580
HERC1
CHC
p-HSP27
Control
SB203580
HERC1
p-HSP27
Control
SB203580
HERC1
CHC
HSP27
Control
SB203580
Control
SB203580
p-HSP27
p38
Control
SB203580
HERC1
CHC
p-p38

Fig. 4

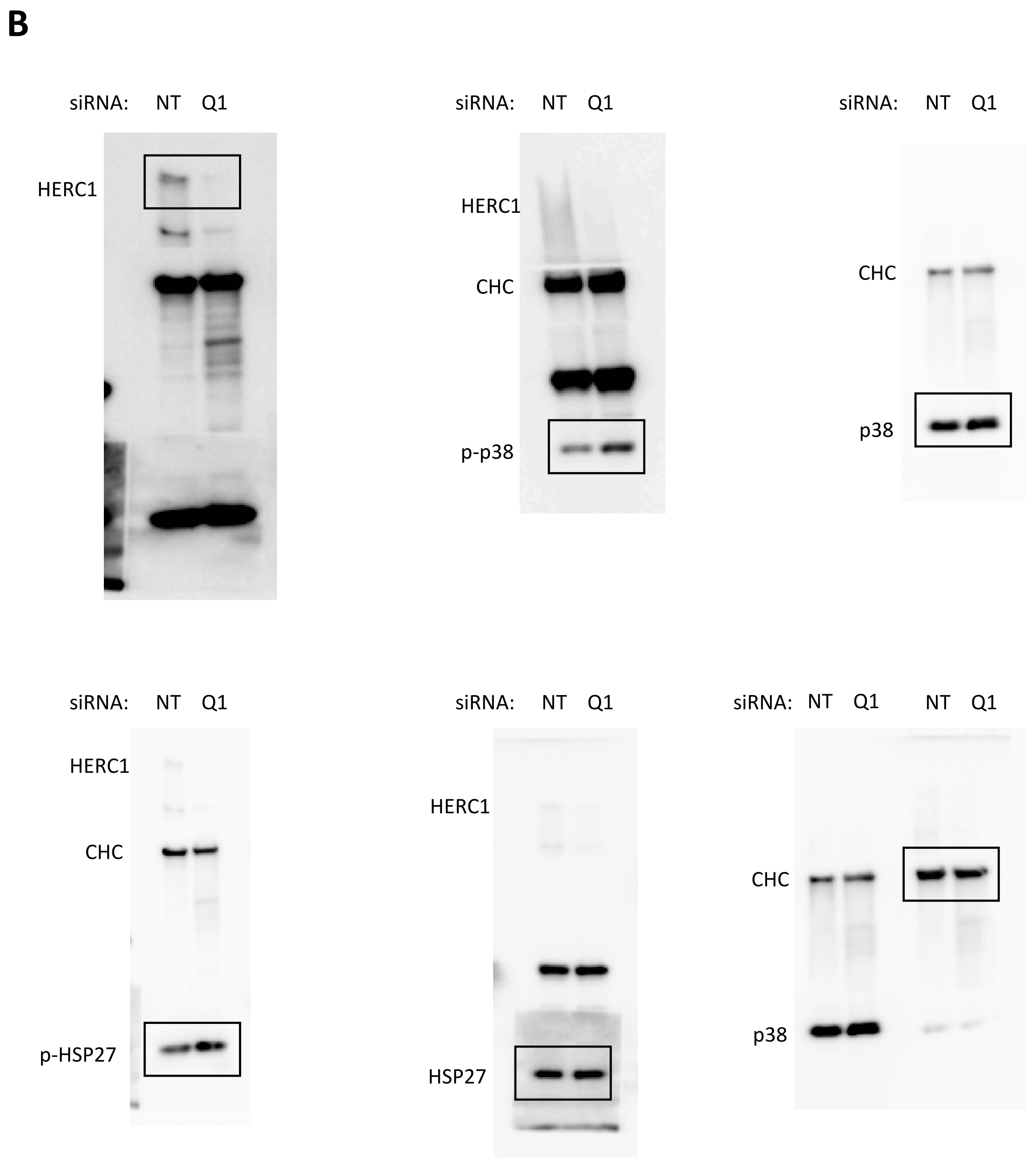
B
siRNA: NT Q1
HERC1
siRNA: NT Q1
HERC1
CHC
p-p38
siRNA: NT Q1
CHC
p38
siRNA: NT Q1
HERC1
CHC
p-HSP27
siRNA: NT Q1
HERC1
HSP27
siRNA: NT Q1 NT Q1
CHC
p38

Fig. 5

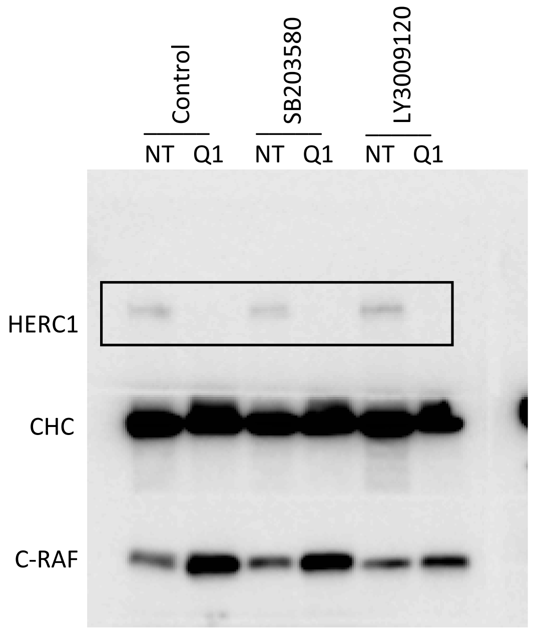
Control
SB203580
LY3009120
NT Q1 NT Q1 NT Q1
HERC1
CHC
C-RAF

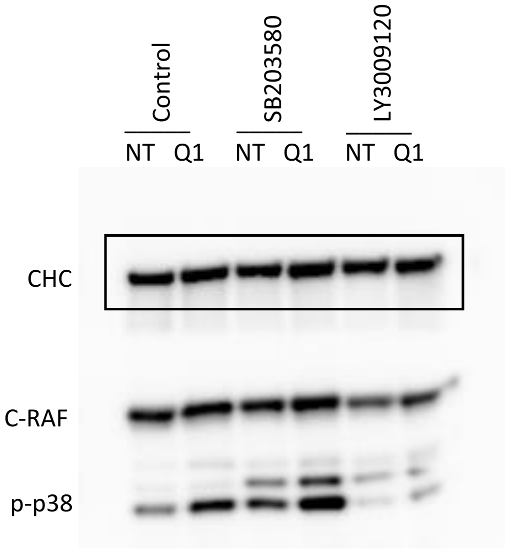
Control
SB203580
LY3009120
NT Q1 NT Q1 NT Q1
CHC
C-RAF
p-p38

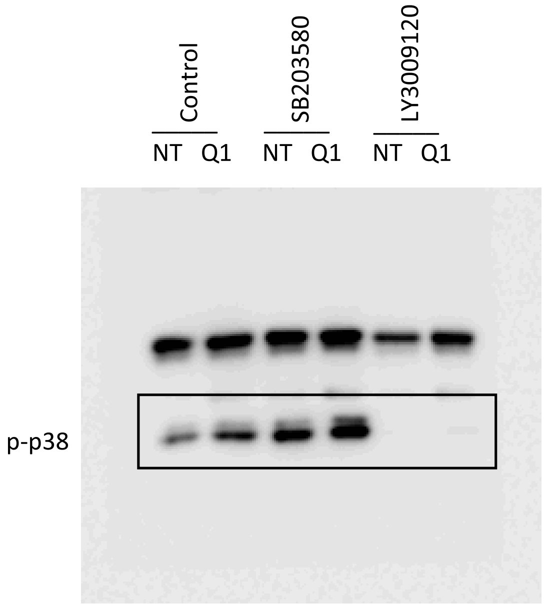
Control
SB203580
LY3009120
NT Q1 NT Q1 NT Q1
p-p38

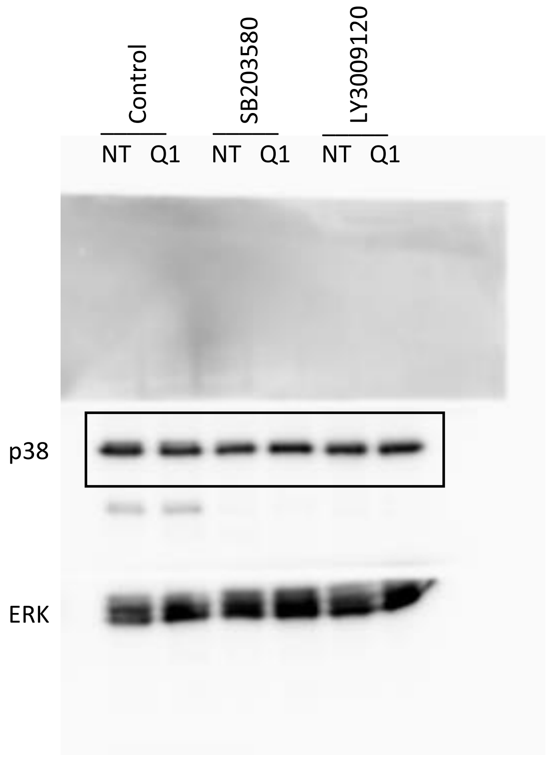
Control
SB203580
LY3009120
NT Q1 NT Q1 NT Q1
p38
ERK

Fig. 5

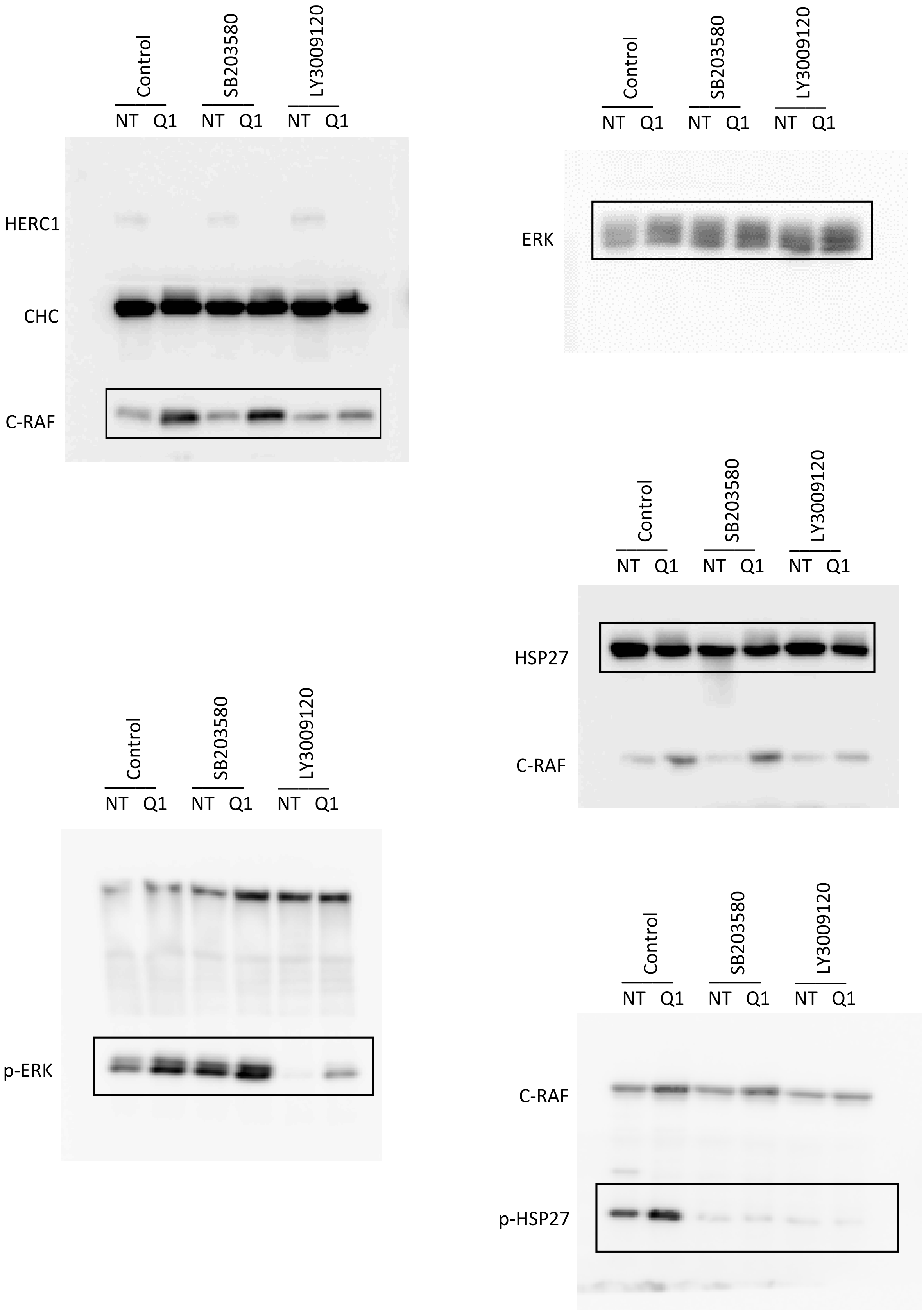

Control
SB203580
LY3009120
NT Q1 NT Q1 NT Q1
HERC1
CHC
C-RAF
ERK
HSP27
p-ERK
p-HSP27

Fig. 6

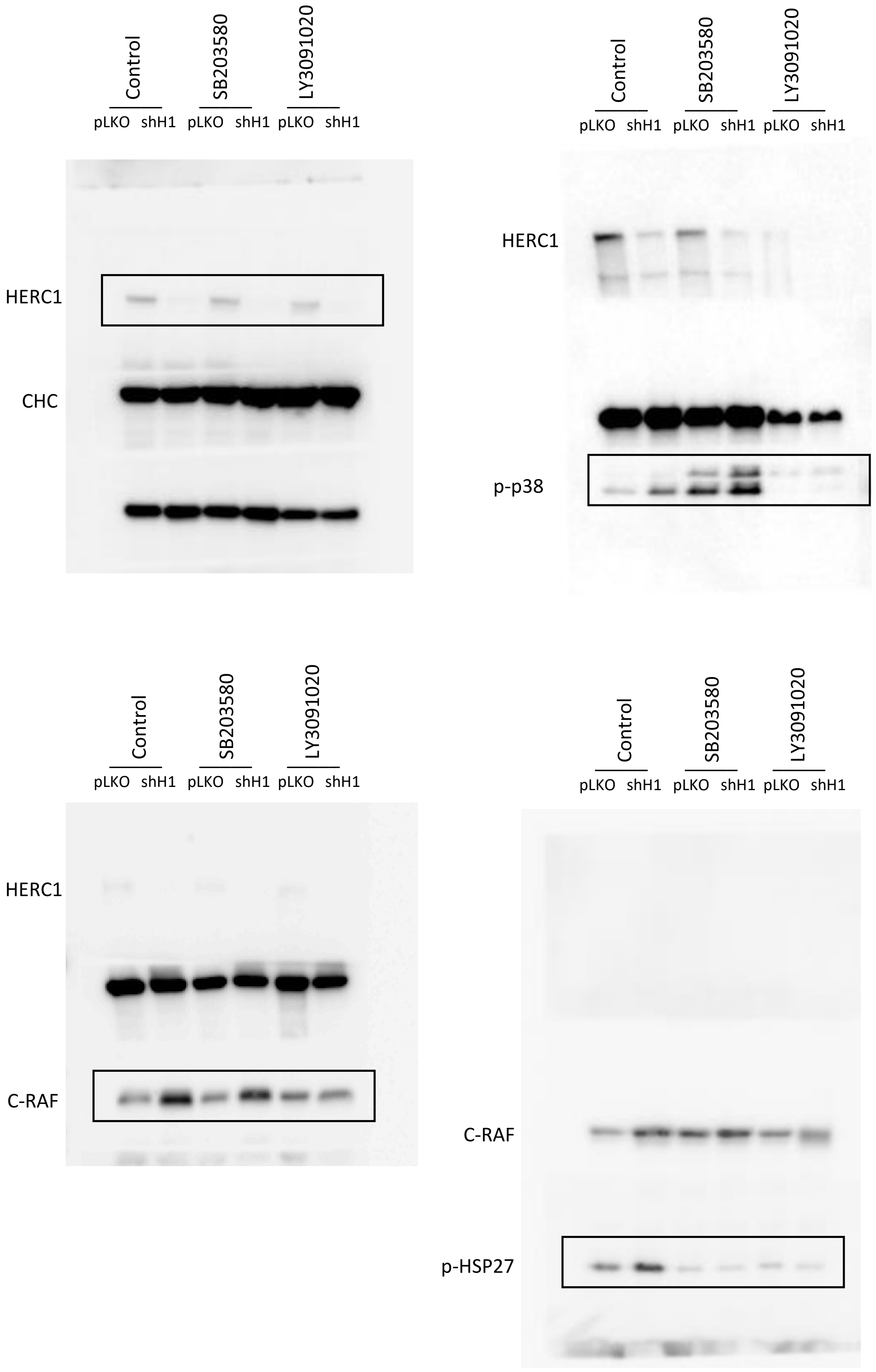
Control
SB203580
LY3091020
pLKO shH1 pLKO shH1 pLKO shH1
HERC1
CHC
p-p38
C-RAF
p-HSP27

Fig. 6

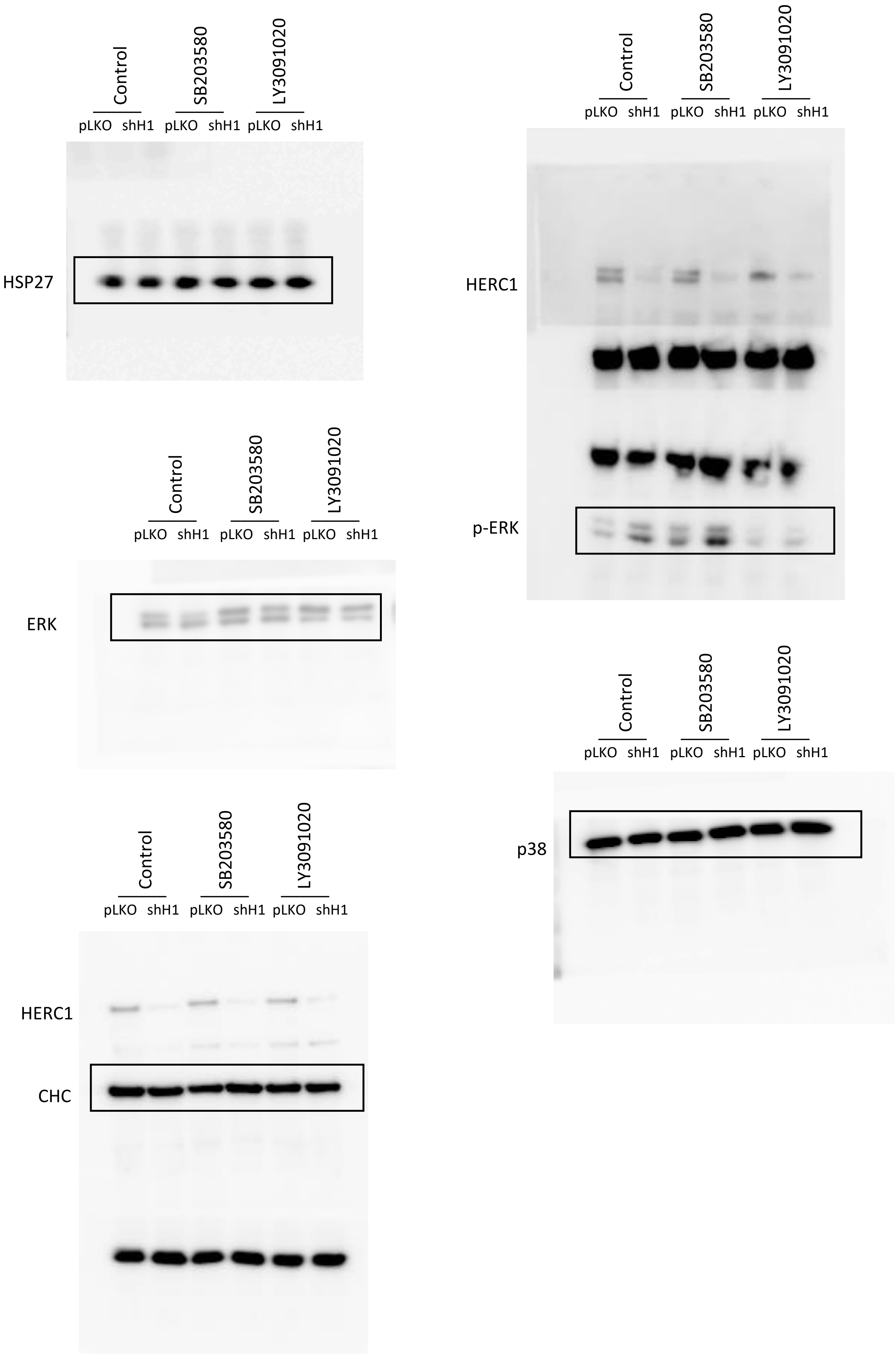
Control
SB203580
LY3091020
pLKO shH1 pLKO shH1 pLKO shH1
HSP27
HERC1
p-ERK
ERK
p38
CHC

Fig. 7

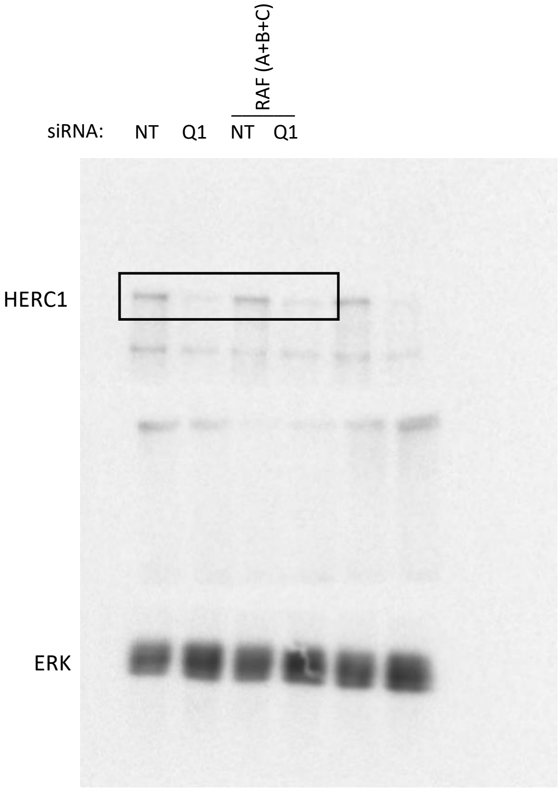
RAF (A+B+C)
siRNA: NT Q1 NT Q1
HERC1
ERK

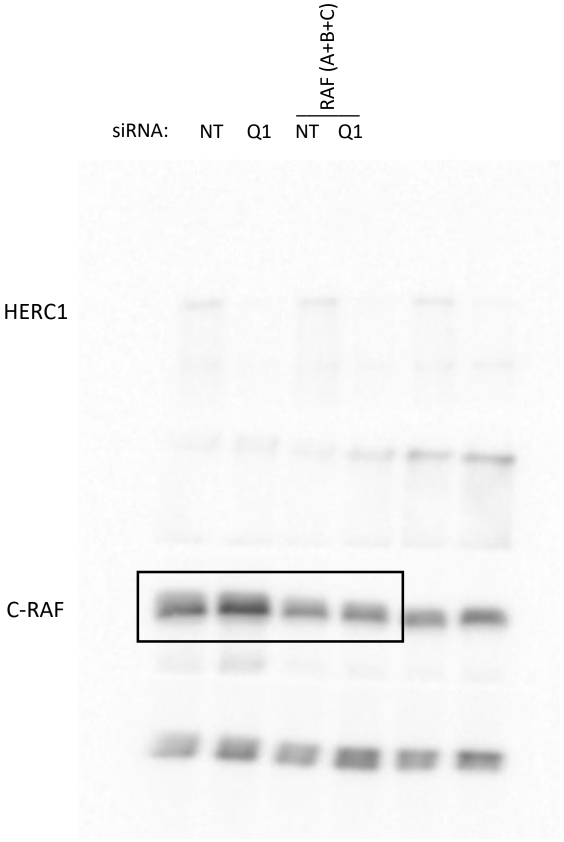
RAF (A+B+C)
siRNA: NT Q1 NT Q1
HERC1
C-RAF

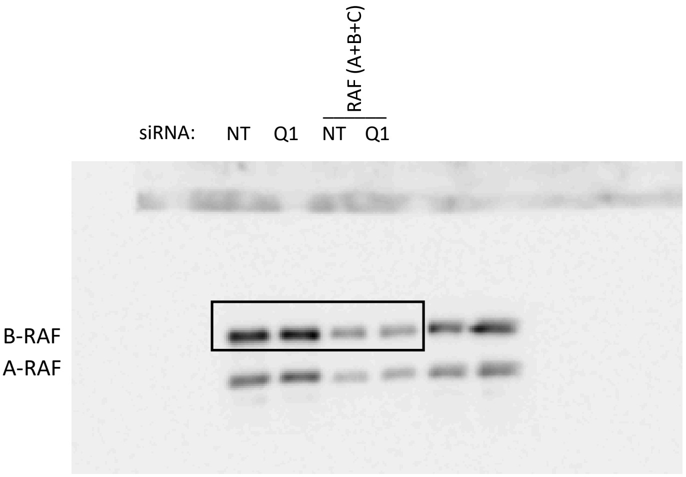
RAF (A+B+C)
siRNA: NT Q1 NT Q1
B-RAF
A-RAF

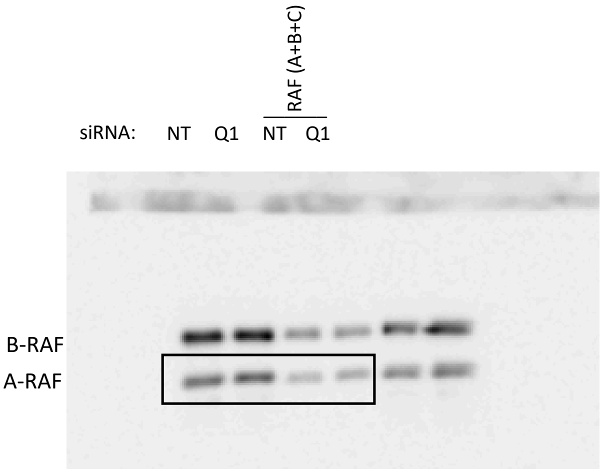
RAF (A+B+C)
siRNA: NT Q1 NT Q1
B-RAF
A-RAF

Fig. 7

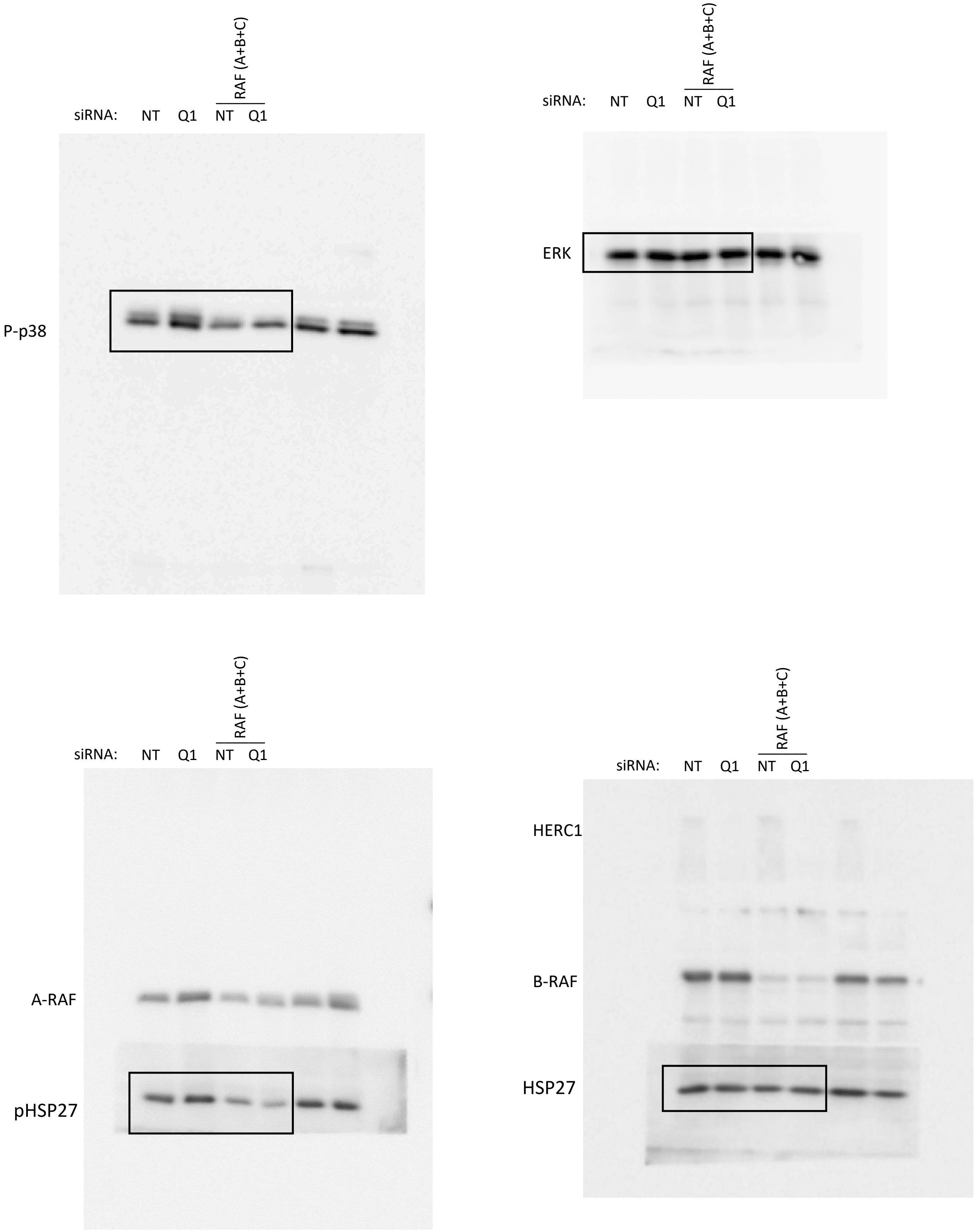
siRNA:
NT
Q1
RAF (A+B+C)
NT
Q1
P-p38
ERK
A-RAF
pHSP27
HERC1
B-RAF
HSP27

Fig. 7

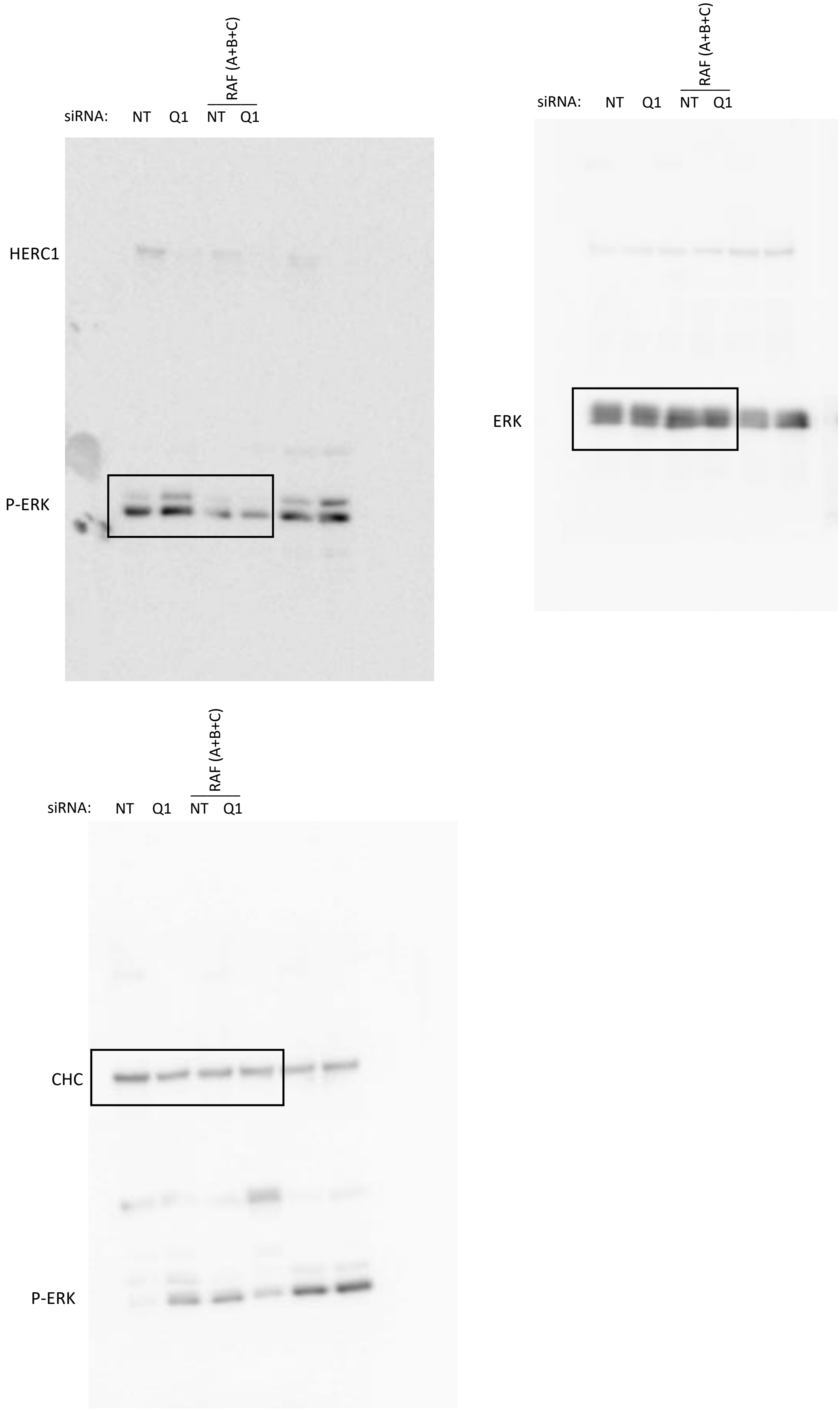

siRNA: NT Q1 NT Q1
RAF (A+B+C)
HERC1
P-ERK
siRNA: NT Q1 NT Q1
RAF (A+B+C)
ERK
siRNA: NT Q1 NT Q1
RAF (A+B+C)
CHC
P-ERK
